# Central amygdalar PKCδ neurons mediate fentanyl withdrawal

**DOI:** 10.1038/s41386-025-02298-7

**Published:** 2025-12-19

**Authors:** Lisa M. Wooldridge, Jacqueline W. K. Wu, Adrienne Y. Jo, Morgan Zinn, Angela M. Lee, Malaika Mahmood, Savanna A. Cohen, Nora M. McCall, Corinna S. Oswell, Sophie A. Rogers, Gregory Corder

**Affiliations:** 1 Department of Psychiatry, Perelman School of Medicine, University of Pennsylvania, Philadelphia, PA USA; 2 Department of Neuroscience, Perelman School of Medicine, University of Pennsylvania, Philadelphia, PA USA; 3 Department of Anesthesiology and Critical Care, Perelman School of Medicine, University of Pennsylvania, Philadelphia, PA USA

**Keywords:** Neuroscience, Cellular neuroscience

## Abstract

Aversion to opioid withdrawal is a significant barrier to achieving lasting opioid abstinence. The central amygdala (CeA), a key brain region for pain, threat-detection, autonomic engagement, and valence assignment, is active during opioid withdrawal. However, the role of molecularly distinct CeA neural populations in withdrawal remains underexplored. Here, we investigated the activity dynamics, brain-wide connectivity, and functional contribution of Protein Kinase C-delta (PKCδ)-expressing neurons in the CeA lateral capsule (CeLC^PKCδ^) during fentanyl withdrawal in mice. Mapping activity-dependent gene expression in CeLC^PKCδ^ neurons revealed a highly withdrawal-active subregion in the anterior half of the CeA. Fiber photometry calcium imaging showed that opioid-naïve CeLC^PKCδ^ neurons respond to salient noxious and startling stimuli. In fentanyl-dependent mice, naloxone-precipitated withdrawal increased spontaneous neural activity and enhanced responses to noxious stimuli. Chronic inhibition of CeLC^PKCδ^ neurons throughout fentanyl exposure, via viral overexpression of the potassium channel Kir2.1, attenuated withdrawal symptoms in fentanyl-dependent mice. Lastly, we identified putative opioid-sensitive inputs to CeLC^PKCδ^ neurons using rabies-mediated monosynaptic circuit tracing and color-switching tracers to map mu-opioid receptor-expressing inputs to the CeLC. Collectively, these findings suggest that the hyperactivity of CeLC^PKCδ^ neurons underlies the somatic signs of fentanyl withdrawal, offering new insights into the amygdala cell-types and circuits involved in opioid dependence.

## Introduction

Opioid Use Disorder (OUD) and its associated overdose deaths remain a leading cause of the ongoing overdose crisis in the United States [1, 2]. Prolonged opioid exposure produces physical dependence, such that cessation of mu-opioid receptor (MOR) agonists or administration of MOR antagonists elicits a highly aversive withdrawal syndrome [3, 4]. Severe withdrawal is among the most frequently reported subjective barriers to maintaining opioid abstinence [3, 5, 6]. An improved understanding of how physical dependence and withdrawal engage the brain circuits that encode aversive states may therefore be critical to developing strategies that make abstinence more tolerable and reduce the risk of relapse and accidental overdose.

The central nucleus of the amygdala (CeA) is a key neural circuit within the brain pathways for aversive processing [7–18]. The CeA coordinates diverse and rapid avoidance, escape, affective, and autonomic responses to potentially threatening or harmful stimuli, helping animals avoid danger and injury [11, 19, 20]. Indeed, antagonist-precipitated opioid withdrawal increases neural activity markers in the CeA, including the immediate early gene FOS [21–23] and calcium (Ca²⁺) activity [24, 25]. Importantly, growing evidence suggests that the CeA operates not as a single unit, but through multiple functionally, transcriptomically, and anatomically distinct cell-types with divergent responses to stimuli and often opposing effects on behavior [13, 16, 17, 26–36]. While recent work increasingly illustrates the diversity of CeA neurons, how these distinct cell-types may uniquely contribute to heightened CeA activity during opioid withdrawal has not been fully explored.

Protein Kinase C-δ-expressing neurons in the lateral division of the CeA (CeLC^PKCδ^) have emerged as crucial regulators of aversive stimuli processing, particularly in relation to pain and threat detection [13, 14]. In rodents, CeLC^PKCδ^ neurons account for approximately half of CeLC neurons, and receive direct input from both environmental threat-encoding parabrachial nucleus and valence-encoding basolateral amygdala cell-types [30, 33, 37–40]. CeLC^PKCδ^ neurons orchestrate adaptive and maladaptive responses in both the short- and long-term, maintaining negative emotional states in anxiety-induced anorexia and conditioned fear [35, 37, 41, 42] and facilitating hypersensitivity in chronic pain models [10, 13, 14, 16].

More recently, CeLC^PKCδ^ neurons have come under investigation in the context of substance use disorders. Recent work shows that CeLC^PKCδ^ neurons mediate drug use-related decision-making during chronic alcohol use [43, 44] and methamphetamine abstinence [45]. Additionally, these neurons exhibit heightened sensitivity to transcriptomic changes following alcohol dependence, highlighting their vulnerability to drug dependence-induced plasticity [31]. Interestingly, a subpopulation of CeLC^PKCδ^ neurons expresses mu-opioid receptors (MORs), the primary molecular target of exogenous opioids such as fentanyl and morphine [12, 25]. It is unclear, however, whether CeLC^PKCδ^ neurons contribute to the various effects of opioid drugs, making them an important and compelling target for further investigation in understanding the neural circuits involved in opioid dependence and withdrawal.

While the CeA is known to be involved in opioid withdrawal—due to its central role in threat detection, autonomic regulation, and negative affective state maintenance—the specific contributions of distinct neural populations, such as PKCδ-expressing neurons, are not fully understood. This study aims to fill this gap by investigating whether CeLC^PKCδ^ neurons contribute to the neural and behavioral effects of opioid withdrawal.

## Materials and methods

For additional experimental details, see Supplementary Materials.

### Animals

All experiments described here were approved by the University of Pennsylvania Institutional Animal Care and Use Committee and performed in accordance with National Institute of Health (NIH) guidelines for animal research. Male and female *Prkcd*-Cre or *Oprm1*-Cre transgenic mice were bred in-house and maintained at heterozygosity. Mice were housed in a temperature- and humidity-controlled vivarium on a 12 h:12 h reverse light/dark cycle (lights off at 9:30 AM). All experimental procedures took place under red light between 10:30 AM-6:30 PM.

### Experimental model of fentanyl physical dependence

**Fig. 1:**
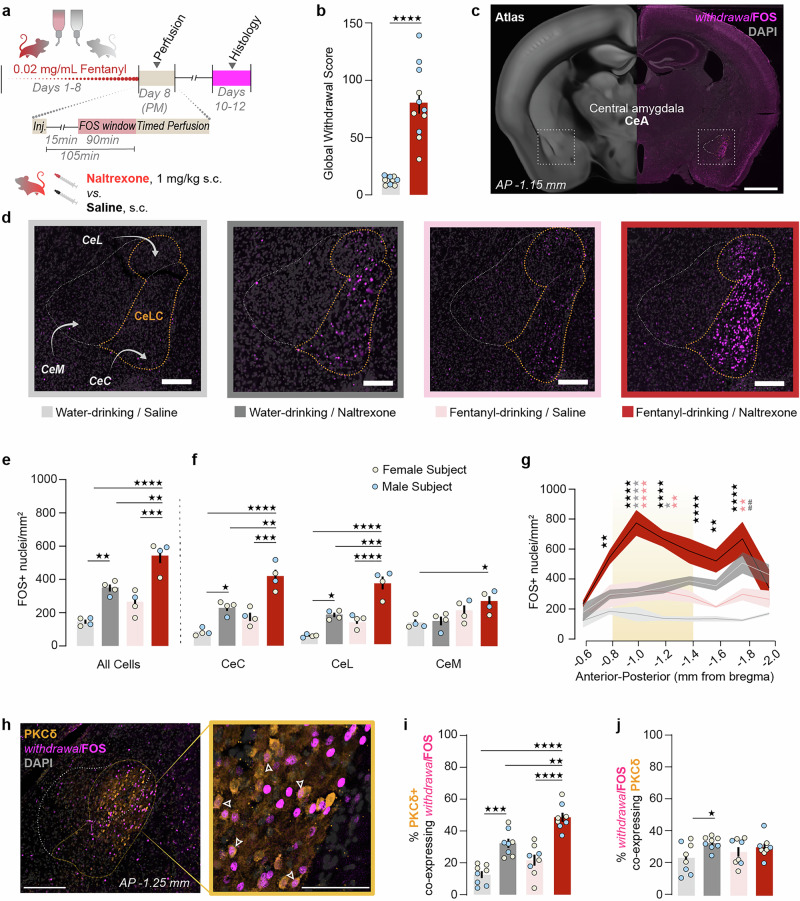
Fentanyl withdrawal increases FOS expression in CeLC^PKCδ^ neurons. **a** Experimental timeline. **b** Global withdrawal scores of water-drinking (n = 5F/5M) and fentanyl-drinking (n = 5F/6M) mice treated with 1 mg/kg naltrexone. Mann-Whitney test, ****p < 0.0001. Bars/error bars are mean ± SEM; individual points represent data from a single mouse (blue: male, tan: female). **c** Representative images of FOS immunoreactivity in the forebrain in fentanyl-drinking mice treated with 1 mg/kg naltrexone (i.e., withdrawal FOS). Scale bar: 1 mm. **d** Representative 20×-magnified z-stack images of FOS immunoreactivity under each condition. Light gray: water-drinking/saline; dark gray: water-drinking/naltrexone; pink: fentanyl-drinking/saline; dark red: fentanyl-drinking/naltrexone. Scale bars: 250 μm. **e** Fentanyl-drinking/naltrexone mice (i.e., withdrawing mice; n = 2F/2M) had significantly higher densities of FOS+ nuclei in the CeA than the mice in any other experimental condition (water/saline: n = 2M, 2F; water/naltrexone: n = 3F/1M; fentanyl/saline: n = 3F/1M). Two-way ANOVA with Bonferroni’s correction, significant effect of drinking condition (F(1,12) = 24.65, *p* = 0.0003) and naltrexone condition (F(1,12) = 59.59, *p* < 0.0001). Bars/error bars are mean ± SEM; individual points represent data for a single mouse (blue: male, tan: female). **p < 0.01; ***p < 0.001; ****p < 0.0001. **f** Fentanyl-drinking/naltrexone mice (i.e., withdrawal mice) had significantly higher densities of FOS+ nuclei in the CeC (left) and the CeL (middle) subnuclei of the CeA than mice in other experimental conditions, but only a higher density than water-drinking/saline mice in the CeM subnucleus (right). Water-drinking/naltrexone mice also had significantly higher densities of FOS+ nuclei in the CeC and CeL compared to water-drinking/saline mice. CeC: Two-way ANOVA with Bonferroni’s correction, significant effect of drinking condition (F(1,12) = 28.68, *p* = 0.0002) and naltrexone condition (F(1,12) = 53.61, *p* < 0.0001). CeL: Two-way ANOVA with Bonferroni’s correction, significant effect of drinking condition (F(1,12) = 34.65, *p* < 0.0001), naltrexone condition (F(1,12) = 63.01, *p* < 0.0001), and naltrexone x drinking interaction (F(1,12) = 5.262, *p* = 0.0406). CeM: Two-way ANOVA with Bonferroni’s correction, significant effect of drinking condition (F(1,12) = 11.78, *p* = 0.005). *p* < 0.05; **p < 0.01; *p* < 0.001; *p* < 0.0001. **g** Fentanyl withdrawal is associated with significantly more FOS+ nuclei in the CeA than any control conditions at AP −1.0 mm and AP −1.2 mm from bregma. Yellow shading: approximate region targeted by viral vector injections in Figs. 2–5. Three-way ANOVA with Bonferroni’s correction, significant effect of coordinate (F(7,96) = 7.103, *p* < 0.0001), drinking condition (F(1,96) = 63.54, *p* < 0.0001), naltrexone condition (F(1,96) = 153.1, *p* < 0.0001), coordinate x drinking interaction (F(7,96) = 3.382, *p* = 0.0029), and coordinate × naltrexone interaction (F(7,96) = 2.882, *p* = 0.0089). Asterisks: fentanyl-drinking/naltrexone vs water-drinking/saline (black); vs. water-drinking/naltrexone (gray); vs. fentanyl-drinking/water (pink); hash marks: water-drinking/naltrexone vs. water-drinking/saline (gray). Points and area fill represent mean values ± SEM. *p < 0.05; **p < 0.01; ***p < 0.001; ****p < 0.0001. **h** FOS immunoreactivity colocalizes with PKCδ immunoreactivity in the CeLC. Arrowheads: PKCδ+/FOS+ cells. Scale bars: large image 250 μm, inset 100 μm. **i** Significantly more PKCδ+ neurons co-express FOS during withdrawal than during any other condition. Two-way ANOVA, significant effect of drinking condition (F(1,28) = 19.82, *p* = 0.0001) and naltrexone (F(1,28) = 66.46, *p* < 0.0001). **j** Significantly more FOS+ neurons co-express PKCδ following a naltrexone injection in naïve mice compared to water-drinking/saline mice. Water-drinking/saline: n = 4m,4f; water-drinking/naltrexone: n = 4m, 4f; fentanyl-drinking/saline: n = 3m, 5f; Fentanyl-drinking/naltrexone: n = 4m, 4f. Two-way ANOVA, significant effect of injection (F(1,28) = 5.384, *p* = 0.0278). *p = 0.0374.

On day 0, the water of fentanyl-drinking mice was replaced with animal facility tap water treated with 0.02 mg/mL fentanyl citrate. Water-drinking control mice drank untreated facility tap water from identical bottles. After 8 days, all bottles were replaced with standard animal facility water bottles containing untreated water. Withdrawal was assessed either immediately following opioid receptor antagonist administration (Figs. 1 and 3) or 20–24 h after the replacement of fentanyl-treated water (Fig. 4).

### Stereotaxic surgery

Adult mice were anesthetized with isoflurane gas in oxygen and mounted onto a stereotaxic frame. 250–500 nL of AAV or RABV*d*G viral vectors were infused at a rate of 100–125 nL/min into the right CeLC (coordinates from Bregma: AP −1.05 mm or −1.10 mm, ML +2.78 mm, DV −4.82 mm). For fiber photometry experiments, an optical fiber (Doric Lenses) was placed 0.2 mm above the targeted injection site immediately after the viral injection. At the completion of experiments, mice were perfused. For photometry experiments, GCaMP6f signal in the CeA was then amplified using immunohistochemistry. Data from mice without on-target virus expression and/or fiber placement were excluded from analysis.

### *withdrawal*FOS induction and immunohistochemistry

Fentanyl- and water-drinking mice were injected with naltrexone (1 mg/kg, s.c.) or saline in their homecage 105 min prior to transcardial perfusion and brain collection (Fig. 1a). We collected every 3rd 30 μm coronal slice through the expanse of the central amygdala and stained for FOS and PKCδ using immunohistochemistry. FOS+ and/or PKCδ+ neurons were quantified bilaterally in each of the CeA subnuclei of one slice per 0.2 mm.

### In vivo fiber photometry

Optical recordings of GCaMP6f fluorescence were acquired using an RZ10x fiber photometry detection system (Tucker-Davis Technologies). All recordings utilized both 460 nm and 405 nm LED-generated light, filtered through a fluorescence minicube and passed through a mono fiber-optic patch cord. The power output at the tip of the patch cable was adjusted daily to ~50 μW for the 460 nm channel (calcium-dependent signal), and to ~15 μW for the 405 nm channel (isosbestic control). Synapse software was used to control the equipment and collect data.

**Fig. 2:**
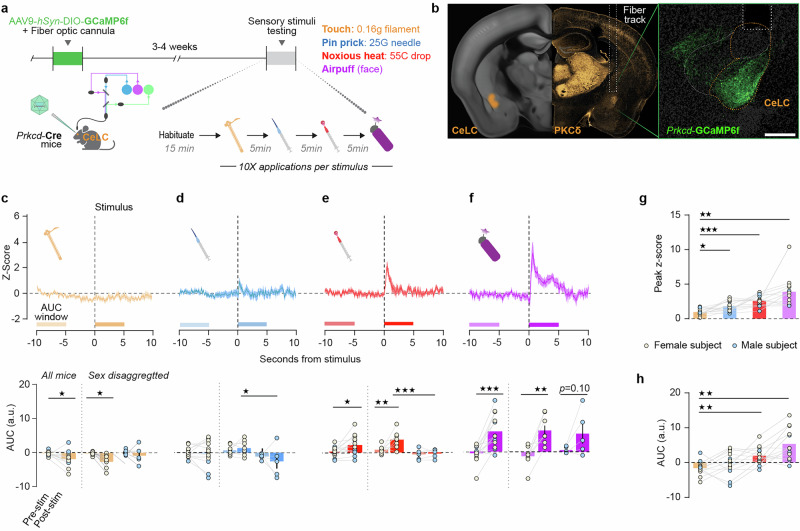
CeLC^PKCδ^ neurons in opioid-naïve mice respond to noxious aversive and non-noxious aversive stimuli. **a** Experimental timeline. **b** Left: PKCδ immunoreactivity and approximate location of fiber optic cannula in the targeted area of the right hemisphere. Right: expression of AAV9-hsyn-DIO-GCaMP6f in the CeLC of a *Prkcd*-Cre mouse, with a fiber optic positioned approximately 200 μm dorsal to the injection site. Scale bar: 250 μm. **c-f **Top: Peri-stimulus time histogram of normalized dF/F (z-score) from −10 to 10 s from the moment of stimulus application, following application of **c** a 0.16 g von Frey filament (innocuous light touch); **d** pinprick with a 25-gauge needle (noxious pinprick); **e** a 55 °C hot water drop (noxious hot water); and **f** an aversive, but non-noxious, airpuff delivered to the side of the face contralateral to virus injection and fiber (aversive airpuff). Traces represent averages of 10 trials/mouse for each stimulus, averaged across all mice (n = 13; 8F/5M). Bottom: Area under the z-score curve from 10 to 5 s prior to stimulus application vs. from 0 to 5 s after stimulus application for all mice (left), female mice only (tan dots, middle), and male mice only (blue dots, right). **c** Compared to pre-stimulus baseline, innocuous light touch was associated with a small decrease in the AUC (Paired t-test, t = 2.663, *p = 0.0207), which was driven by significant decreases primarily in female mice. Two-way Repeated-Measures ANOVA with Bonferroni’s correction, significant effect of timepoint (F(1,11) = 5.570, *p* = 0.0378). Bars represent mean z-score ± SEM; dots represent individual points. *p = 0.0329. **d** Noxious pinprick did not significantly affect AUC (Paired t-test), but female mice showed significantly higher post-stimulus AUCs than male mice. Two-way ANOVA with Bonferroni’s correction, significant effect of sex (F(1, 11) = 6.189, *p* = 0.0302). *p = 0.0232. **e** Noxious hot water was associated with a significant increase in AUC (Paired t-test, t = 2.590, *p = 0.0237), driven by a significant increase between timepoints primarily in female mice (**p = 0.0046) and significantly greater post-stimulus AUCs in female compared to male mice (***p = 0.0005). Two-way Repeated Measures ANOVA with Bonferroni’s correction, significant effect of timepoint (F(1, 11) = 5.570, *p* = 0.037) and sex (F(1, 11) = 13.14, *p* = 0.0040). **f** Aversive airpuff was associated with a significant increase in AUC (Paired t-test, t = 4.733, ***p = 0.0005), which was driven by significant increases in female mice and a trend towards significant increases in male mice. Two-way Repeated Measures ANOVA with Bonferroni’s correction, significant effect of timepoint (F(1, 11) = 19.09, *p* = 0.0011). **p = 0.0025. Lines and area fill represent mean values of 10 trials/subject, averaged across all subjects, ±SEM (n = 8 females and 5 males). **g** Pinprick, noxious hot water, and aversive non-noxious airpuff produced a significantly higher peak than innocuous light touch (One-way repeated measures ANOVA with Bonferroni’s correction, significant effect of stimulus (F(1.333, 15.99) = 58.07, *p* < 0.0001) and significant effect of subject (F(12,36) = 15.80, *p* < 0.0001). *p = 0.0263; ***p = 0.002; **p = 0.0034. **h** Compared to innocuous light touch, noxious hot water, and aversive airpuff produced a significant increase in the AUC. Repeated measures one-way ANOVA with Bonferroni’s correction, significant effect of treatment (F(2.50, 30.96) = 11.35, *p* < 0.0001).*p = 0.0114, ***p = 0.0009.

**Fig. 3:**
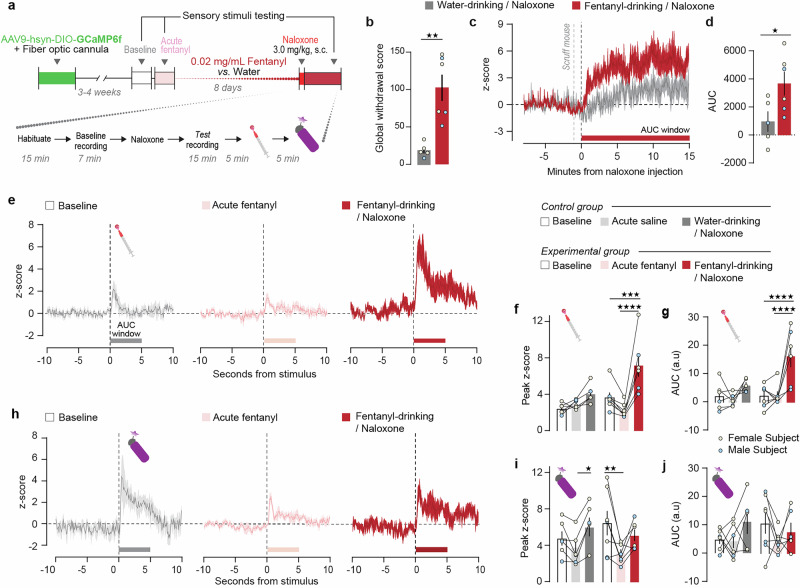
CeLC^PKCδ^ neurons are hyperactive and hypersensitive during fentanyl withdrawal. **a** Experiment timeline. After baseline sensory testing (Fig. 2), mice were separated into a control group that remained opioid-naïve prior to naloxone administration on day 8 (control group; n = 4F/2M) and an experimental group that received all fentanyl treatments (experimental group; n = 4 F/3M). **b** Fentanyl-drinking mice exhibited higher global withdrawal scores following naloxone administration compared to water-drinking control mice. (Unpaired t-test, t = 4.228), **p = 0.0022. **c** Peri-injection time histogram of fluorescence before, during, and after an injection of 3 mg/kg naloxone in control, naïve animals (dark gray) and fentanyl-dependent animals (red). Red bar: AUC interval in (**d**). Traces are averaged across all subjects (n = 5 (control group; 1 male excluded) and n = 6 (experimental group; 1 female excluded)). **d** The net AUC for the 15 min after naloxone injection is significantly higher for fentanyl-drinking vs. naïve mice (Unpaired t-test, t = 2.663, *p = 0.0207). **e** Peri-stimulus time histogram from 10 s prior to 10 s following application of a noxious hot water drop for the fentanyl-dependent group at baseline (light gray trace), after 0.2 mg/kg fentanyl prior to drinking (pink), and during fentanyl withdrawal (red). Lines and area fill represent mean values ± SEM of five trials averaged across subjects. **f** The peak of the fluorescence response in the 5 s following application of noxious hot water was significantly higher during fentanyl withdrawal than at baseline or after an acute injection of fentanyl in the experimental group. Mixed-effects analysis with Bonferroni’s adjustment, main effect of treatment (F(2,20) = 19.75; *p* < 0.0001) and group × treatment interaction (F(2,20) = 7.295, *p* = 0.0042) ***p = 0.0001, ****p < 0.0001. **g** 5-s post hot water AUC was significantly higher during withdrawal than during other conditions. Mixed-effects analysis with Bonferroni’s correction, significant effect of treatment (F(2,31) = 12.68, *p* < 0.0001), group (F(1,31) = 5.791, *p* = 0.0223), and group x treatment interaction (F(2,31) = 3.367, *p* = 0.0475). ****p < 0.0001. **h** Peri-stimulus time histogram from 10 s prior to 10 s following application of a noxious hot water drop for the fentanyl-dependent group. Line and area fill represent mean ± SEM value of five trials averaged across subjects. **i** Acute fentanyl injection decreased the peak of the fluorescence response to airpuff relative to baseline in the experimental group (*p* = 0.0038) and relative to naloxone injection in the control group (*p* = 0.0231). (Mixed-effects analysis with Bonferroni’s adjustment, main effect of experimental timepoint (F(2, 20) = 9.110, *p* = 0.0015). **j** No significant changes in 5 s AUC during any condition in either group.

On the baseline test day, mice received 10 trials each of the 0.16 g von Frey hair, 25G-needle pinprick, 55 °C hot water drop (all delivered to the left hindpaw), and 1-s airpuff (delivered 1 cm from the left cheek), with a 90 s ITI and a 5 min recording break between stimulus types (Fig. 2a). During the naloxone-precipitated withdrawal test, Ca^2+^ activity was recorded 7 min prior to, during, and 15 min after drug treatment; after a 5-min rest, mice received 5 trials each of a 55 °C hot water drop and a 1-s airpuff (Fig. 3a). Light delivery was controlled by and data was collected in Synapse software. A TTL pulse was delivered to the recording system by means of a custom-made trigger button to timestamp stimulus application and drug injection.

Data from the Synapse software was processed using an independent deployment of pMAT software [46] and MATLAB to extract dF/F and z-scored mean traces. The peak amplitude post-stimulus, and areas under the curve pre- and post-stimulus or post-drug injection, were calculated from these traces in Prism (Graphpad, v9).

### Viral-mediated overexpression of Kir2.1

**Fig. 4:**
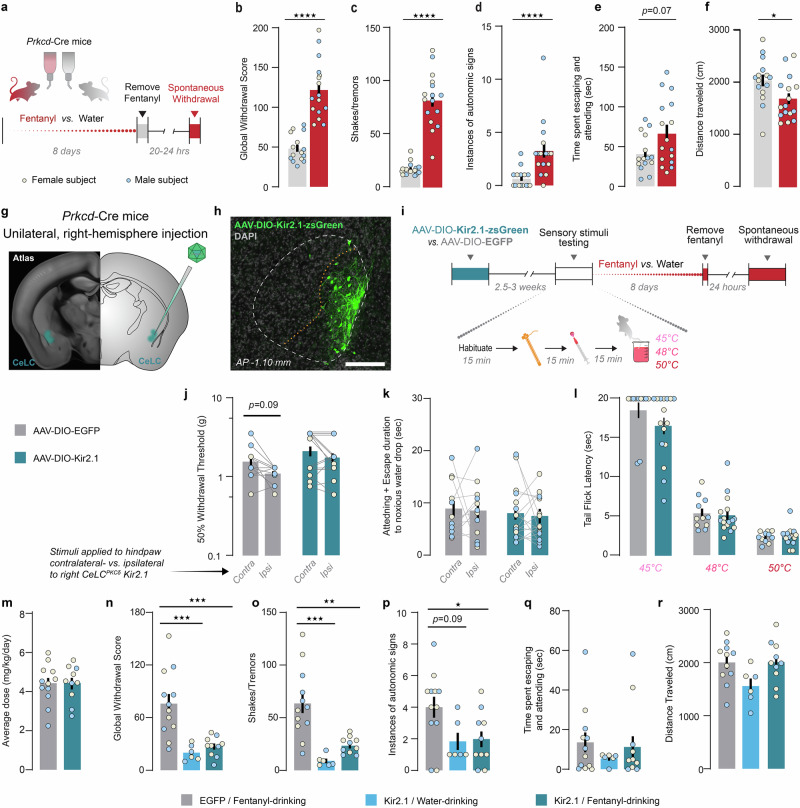
Chronic inhibition of CeLC^PKCδ^ neurons prevents fentanyl withdrawal without impacting pain sensitivity. **a**–**f** Characterization of spontaneous withdrawal behavioral phenotype in *Prkcd-*Cre mice. **a** Experimental timeline. **b** Fentanyl-drinking mice exhibited significantly higher global withdrawal scores 20–24 h after the removal of fentanyl compared to water-drinking mice. Unpaired t-test (t = 7.916). ****p < 0.0001. Bars represent mean ± SEM; dots are individual subjects’ data points. Water-drinking: n = 7F/8M, Fentanyl-drinking: n = 8F/8M Fentanyl-drinking mice exhibited significantly more **c** shakes and tremors (Unpaired t-test, ****p < 0.0001) and **d** autonomic signs (Mann-Whitney test, U = 27.50, ****p < 0.0001). **e** Fentanyl-drinking mice did not exhibit significantly higher time engaging in affective responses during withdrawal compared to water-drinking mice (Mann-Whitney test). **f** Fentanyl-drinking mice traveled a significantly shorter distance than water-drinking mice during the spontaneous withdrawal observation period (Unpaired t-test, t = 2.293, *p = 0.0293). **g**–**r** Effects of Kir2.1 overexpression in CeLC^PKCδ^ neurons. **g** Experimental approach. **h** Expression of AAVDJ-CMV-DIO-Kir2.1-zsGreen in the CeLC of *Prkcd-*Cre mice. Scale bar: 250 μm. **i** Experimental timeline and testing procedure for sensory testing. **j** We detected a main effect of stimulation side (Two-way ANOVA with Bonferroni’s correction, main effect of stimulation side (F(1, 25) = 7.274, *p* = 0.0123), effect of subject (F(25,25) = 3.721, *p* = 0.0008) but not viral condition on 50% withdrawal thresholds in the von Frey Up-Down assay. Mice had slightly (but not significantly) higher 50% withdrawal thresholds on the hindpaw contralateral to the hemisphere of virus injection compared to the ipsilateral paw, but this was true only for EGFP-expressing mice. Gray bars: EGFP-expressing mice; Blue/teal bars: Kir2.1-expressing mice. Bars represent mean values ± SEM; gray points represent individual subjects’ values. EGFP: n = 6F/6M. Kir2.1: n = 9F/7M. **k** Kir2.1 overexpression does not affect the time spent engaging in affective responses to the application of a noxious hot water drop, regardless of which hindpaw is stimulated (Two-Way repeated measures ANOVA). **l** Kir2.1 overexpression does not impact the latency for mice to withdraw tails from hot water at 3 different temperatures (Two-Way Repeated Measures ANOVA with Bonferroni’s correction, main effect of temperature (F(2,54) = 165.9; *p* < 0.0001). **m** Kir2.1-overexpressing mice consumed a similar average daily dose of fentanyl as EGFP-expressing control mice (Unpaired t-test). EGFP + Fentanyl-drinking: n = 6F/6M; Kir2.1 + Fentanyl-drinking: n = 6F/4M. **n** Kir2.1-overexpressing mice (teal) show significant fewer withdrawal signs after fentanyl exposure compared to EGFP-expressing mice (gray), and similar global withdrawal scores to Kir2.1-expressing mice that drank water (light blue; One-way ANOVA with Bonferroni’s correction, F(2,25) = 6.116), *p* = 0.0069; ***p < 0.001). EGFP + Fentanyl-drinking: n = 6F/6M; Kir2.1 + Fentanyl-drinking: n = 6F/4M; Kir2.1 + water-drinking: n = 3F/3M. **o** Kir2.1-expressing, fentanyl-drinking mice and Kir2.1-expressing, water-drinking mice exhibited significantly fewer shakes and tremors compared to EGFP-expressing, fentanyl-drinking mice (One-way ANOVA with Bonferroni’s correction, F(2,25) = 5.854, *p* < 0.0001). **p = 0.001, ***p = 0.0002. **p** Kir2.1 expression significantly reduced the autonomic subscore of fentanyl-dependent mice (One-way ANOVA with Bonferroni’s correction, (F(2,27) = 5.083, *p* = 0.0134; *p = 0.01). **q** Kir2.1 expression did not significantly reduce the time spent engaging in affective behaviors in fentanyl-dependent mice (One-way ANOVA). **r** No significant effect of Kir2.1 overexpression on withdrawal-associated hypolocomotion (One-way ANOVA, F(2,24) = 3.088, *p* = 0.064).

300 nL of AAVDJ-*CMV*-DIO-Kir2.1-zsgreen or AAV5-*hSyn*-DIO-EGFP was injected into the right CeA of male and female *Prkcd*-Cre mice. 2.5–3 weeks later, mice underwent baseline testing to determine values for mechanical and thermal reflexive thresholds and to determine affective responses to thermal stimuli (Fig. 4i). Mice then began the 8-day fentanyl or water drinking procedure. On Day 9, 20–24 h after fentanyl removal, mice were returned to the same testing room. Video recordings taken from underneath the mice were collected in the following 15 min and were scored offline to obtain spontaneous global withdrawal score and distance traveled. Mice were then re-tested on sensory stimuli.

### Monosynaptic rabies tracing

**Fig. 5:**
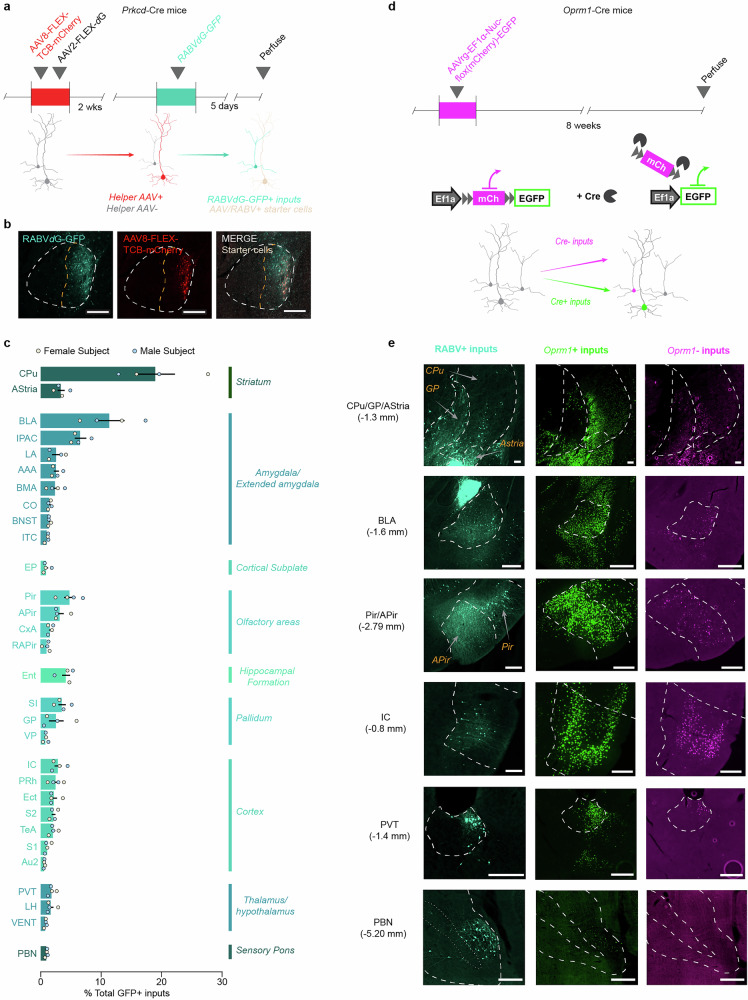
Presynaptic input architecture of opioid-receptor-expressing circuits to CeLC^PKCδ^ neurons. **a** Timeline for the expression of RABV*d*G in the CeA of *Prkcd*-Cre mice. Cre+ cells are initially transfected with Cre-dependent “helper” AAVs containing a modified TVA receptor tagged with mCherry and the rabies glycoprotein. When RABV*d*G-GFP is injected into the CeA 2 weeks later, it can only transfect cells that took up the helper AAVs (“starter cells”; tan). RABV*d*G-GFP moves one synapse back, labeling direct inputs to the “starter cells” with GFP (cyan). **b** Expression of RABV*d*G-GFP and the fluorescently-labeled helper virus AAV8-hsyn-FLEX-TCB-mCherry containing the modified TVA receptor in CeLC^PKCδ^ neurons in the CeA. Scale bars: 250 μm. **c** Top 30 regions identified with the highest densities of inputs to CeLC^PKCδ^ neurons (i.e., RABV*d*G-GFP-labeled cells). Bars represent mean percentage of identified GFP+ cells ± SEM; dots represent values for individual subjects. n = 2F/2M. **d** Experimental timeline for the expression of AAVrg-EF1α-Nuc-flox(mCherry)-EGFP in *Oprm1*-Cre mice. In the presence of Cre, mCherry will be deleted due to the orientation of loxP sites; *Oprm1*-Cre+ cells thus express the EGFP. In the absence of Cre, mCherry is not deleted, and a stop codon downstream of the mCherry prevents the expression of EGFP; *Oprm1*-Cre- cells thus express mCherry. n = 3M. **e** Representative images of RABV*d*G-GFP-labeled (cyan), GFP-labeled Cre+ cells (green), and mCherry-labeled Cre- cells (magenta) in notable input regions. Scale bars: 200 μm. Reported values are percent *Oprm1+* cells and sum of all counted cells in that region across all mice (n = 3M). CPu caudate-putamen (66%, 28119 cells), AStria amygdalostriatal transition area (80%, 4813 cells), BLA basolateral amygdala (78%, 11105 cells), IPAC Interstitial nucleus of the anterior limb of the posterior commissure (74%, 3177 cells), LA lateral amygdala (77%, 4266 cellls), AAA anterior amygdaloid area (60%, 1622 cells), BMA basomedial amygdala (81%, 6262 cells), CO cortical amygdala (71%, 1222 cells), BNST Bed nucleus of the stria terminalis (73%, 1472 cells), ITC intercalated nuclei of the amygdala (72%, 1222 cells), EP endopiriform nucleus (75%, 3559 cells), Pir piriform cortex (65%, 10841 cells), APir postpiriform transition area (78%; 5885 cells), CxA cortex-amygdala transition area (70%, 1407 cells), Ent entorhinal cortex (80%, 9811 cells), SI substantia innominata (67%, 1171), GP Globus pallidus (71%, 3380 cells), VP ventral pallidum (57%, 1051 cells), IC insular cortex (55%, 22630 cells), PRh perirhinal cortex (61%, 3762 cells), Ect ectorhinal cortex (72%, 7204 cells), S2 secondary somatosensory cortex (73%, 4792 cells), TeA temporal association cortex (79%, 5197 cells), S1 primary somatosensory cortex (67%, 6820 cells), AuV/AuD ventral and dorsal auditory cortex (73%, 2024 cells), PVT paraventricular nucleus of the thalamus (75%, 3968 cells), LH lateral hypothalamus (71%, 1902 cells): VENT ventral posterior thalamic nuclear group (72%, 169 cells), PBN parabrachial nucleus (65%, 330 cells).

500 nL of a 1:1 mixture of AAV2-DIO-TCB-mCherry and AAV8-FLEX-RABV*d*G was injected into the right CeLC of male and female *Prkcd*-Cre mice, followed 2 weeks later by 500 nL of RABV*d*G-GFP (Fig. 5a). Five days later, mice were perfused, and consecutive 50 μm slices were collected through the entire brain. Every 2nd 50 μm slice from the central amygdala was imaged to confirm the presence of starter cells. We then selected coordinates of interest from an initial scan of GFP+ cells. Every right hemisphere slice from these coordinates was then imaged. GFP+ input cells on each slice were counted, then aligned to the Kim Lab Unified Anatomical Atlas [47] using the MATLAB-based atlas registration program SHARCQ [48].

### Identification of opioidergic inputs to the central amygdala

250 nL of AAVretrograde-*EF1a*-Nuc-flox(mCherry)-EGFP was injected into the right CeA of male and female *Oprm1-*Cre mice (Fig. 5d). Eight weeks later, mice were perfused, and 50 µm slices were collected every 0.2 mm throughout the brain. The mCherry fluorescent signal was amplified using immunohistochemistry, and then we imaged each region identified in Fig. 5c.

### Statistical analyses

Power analyses were conducted in G*Power [49] at the beginning of each experiment to determine minimum sample sizes and adjusted as needed based on observed effect sizes. Kolgomorov-Smirnoff tests were used to assess the normality of data sets, and statistical tests were then calculated as indicated using Prism Software (Graphpad, v9). Unless otherwise noted, data are presented as the mean ± standard error of the mean, with gray circles representing the observed value for an individual subject.

## Results

### Precipitated fentanyl withdrawal increases cellular activity in CeLC^PKCδ^ neurons

To test whether fentanyl withdrawal increases CeLC^PKCδ^ neuron activity, we induced physical dependence in male and female *Prkcd*-Cre mice by providing 24-h free access to fentanyl-treated water (0.02 mg/mL) for 8 days in the homecage (Fig. 1a). Control mice drank untreated water. Fentanyl-treated mice consumed 3–5 mg/kg/day under this procedure, but did not develop a preference for fentanyl-treated water in a two-bottle choice assay (Fig. S1). On the eighth day, mice received an injection of either naltrexone (1 mg/kg, s.c.) or saline. Naltrexone elicited classic murine withdrawal signs in fentanyl-drinking mice, but not water-drinking mice, confirming the development of physical dependence (Fig. 1b). Global withdrawal scores directly correlated with animals’ fentanyl intake (Fig. S1i, j). After 105 min, brains were collected and immunostained for FOS, an immediate early gene product used as a marker of neural activity. We found a significant effect of drinking treatment and naltrexone administration on the number of FOS⁺ nuclei in the CeA. Specifically, fentanyl-drinking mice that received naltrexone (i.e., withdrawing mice) had significantly higher densities of FOS⁺ nuclei than any other condition (*withdrawal*FOS*;* Fig. 1c–e). Naltrexone also increased FOS+ nucleus density in water-drinking mice, while fentanyl drinking alone did not.

*withdrawal*FOS⁺ nuclei were predominantly localized to the lateral division of the CeA (CeLC) (Fig. 1c, d, f). Withdrawing mice had significantly higher densities of FOS⁺ nuclei than all other groups in both the capsular (CeC) and the lateral subnuclei (CeL) of the CeLC (Fig. 1f). Notably, naltrexone in water-drinking mice also selectively increased FOS⁺ nuclei in the CeLC, suggesting tonic endogenous opioid activity in this region (Fig. 1f). Meanwhile, withdrawing mice exhibited a higher density of FOS+ nuclei only compared to water-drinking, saline-injected mice in the medial division of the CeA (CeM; Fig. 1f). *withdrawal*FOS expression was significantly higher in the CeLC than in the CeM across all fentanyl-drinking, naltrexone-treated mice (Fig. S2a, b).

*withdrawal*FOS was elevated compared to that of untreated controls throughout the anterior-posterior axis, except at the most anterior (−0.6 mm) and posterior (−2.0 mm) poles (Fig. 1g). However, *withdrawal*FOS density was only significantly higher in withdrawing mice than in all experimental groups at −1.0 to −1.2 mm AP. Despite prior studies suggesting a greater role for the right CeA in aversive processing [15, 50–52], we did not observe lateralization of FOS expression (Fig. S2c–g).

Next, we investigated whether withdrawal specifically increased FOS expression in CeLC^PKCδ^ neurons. We repeated the prior study, co-immunostaining for FOS and PKCδ (Fig. 1h). In both sexes, withdrawal increased the proportion of PKCδ neurons that co-express FOS (Figs. 1i and S2i). In water-drinking mice, naltrexone also increased both the proportion of PKCδ neurons co-expressing FOS, as well as the proportion of FOS+ neurons co-expressing PKCδ (Fig. 1i, j), indicating an endogenous opioid tone in the CeA [12, 53].

### CeLC^PKCδ^ neurons are tuned to salient aversive and noxious stimuli

Based on our initial FOS mapping, which indicated increased neural activity from −1.0 to −1.2 mm AP during fentanyl withdrawal (Fig. 1g), we next investigated the temporal dynamics of anterior CeLC^PKCδ^ neurons using in vivo fiber photometry in behaving mice. We expressed the Ca²⁺ indicator GCaMP6f in this region of the CeA of *Prkcd*-Cre mice and placed an optical fiber 0.2 mm above the injection site to measure stimulus-evoked fluorescence changes (Fig. 2a, b). Although several studies demonstrate the role of CeLC^PKCδ^ neurons in generating and responding to aversive states [13, 16, 33, 42, 54], few have directly examined their immediate responses to discrete noxious and aversive stimuli.

To address this, we measured Ca²⁺ responses to four somatosensory stimuli: innocuous light touch (0.16 g von Frey filament), noxious, aversive stimuli (25-gauge sharp pinprick or 55 °C hot water drop), and a non-noxious but aversive stimulus (1-s airpuff; Fig. 2c–f). Innocuous light touch resulted in a small decrease in z-scored Ca²⁺ signal, dropping below baseline ~5 s before the stimulus and remaining suppressed for ~8 s post-stimulus (Fig. 2c). In contrast, all aversive stimuli produced a Ca²⁺ increase (Fig. 2d–h). The signal following noxious pinprick and hot water returned to baseline within ~3 s (Fig. 2d, e), whereas the response to aversive airpuff remained elevated for ~6 s post-stimulus (Fig. 2f). Compared to innocuous light touch, noxious pinprick, noxious hot water drop, and aversive non-noxious airpuff produced a significantly higher peak z-scored Ca²⁺ response in the 5 s following stimulus application (Fig. 2g). Non-normalized dF/F followed the same pattern, though pinprick did not reach significance for this metric (Fig. S3b–f).

We also analyzed the area under the z-scored Ca²⁺ curves (AUC) before and after each stimulus. AUC slightly decreased pre- and post-innocuous light touch (Fig. 2c), while no significant changes were observed for noxious pinprick (Fig. 2d). Both noxious hot water (Fig. 2e) and airpuff (Fig. 2f) significantly increased AUC relative to baseline, with the AUC post-aversive airpuff also significantly greater than post-innocuous light touch (Fig. 2h). These effects were driven by stimulus-induced activity changes in female, but not male, subjects for hot water, and both sexes for airpuff. Non-normalized dF/F followed similar patterns (Fig. S3b–e, g). From these findings, we conclude that in opioid-naïve female mice only, CeLC^PKCδ^ neurons primarily respond to aversive stimuli, and do not differentiate noxious vs. non-noxious aversive stimuli.

We then related stimulus-evoked Ca^2+^ activity to stimulus-evoked behavior (Fig. S3h–n). We found that the Ca^2+^ signal more closely resembles the stimulus applied than it does any specific escape or avoidance response (Fig. S3i–l). This latter finding suggests that CeLC^PKCδ^ neurons more closely encode stimulus content than behavioral response, consistent with their role as input-receiving interneurons.

### Opioid administration and precipitated withdrawal dynamically modulate CeLC^PKCδ^ neuron activity

We next examined how CeLC^PKCδ^ neurons respond to aversive stimuli during fentanyl withdrawal. (Fig. 3a). We rendered the same mice opioid-naïve or dependent using the procedure described in Fig. 1a. On the eighth day of fentanyl consumption, all mice received naloxone (3 mg/kg, s.c.). As expected, fentanyl-drinking mice exhibited significantly higher global withdrawal scores than water-drinking mice following naloxone (Fig. 3b). In opioid-naïve mice, Ca²⁺ activity transiently dropped below baseline following naloxone injection before returning to pre-drug levels within ~10 min (Figs. 3c and S3a). However, in fentanyl-dependent mice, Ca²⁺ activity did not decrease but instead rose sharply during and after naloxone administration, remaining significantly elevated throughout the 15-min recording period (Figs. 3c, d and S4a, b). These findings build on our previous results (Fig. 1), demonstrating a rapid and sustained increase in CeLC^PKCδ^ neuron activity during precipitated opioid withdrawal.

To determine how fentanyl alters CeLC^PKCδ^ responses to aversive stimuli, we compared the Ca²⁺ responses to a noxious hot water drop (Fig. 3e–g) and an aversive airpuff (Fig. 3h–j) under baseline conditions (Fig. 2), after an acute fentanyl injection (0.2 mg/kg, s.c.) while mice were opioid-naïve, and during precipitated withdrawal. An experimental group received both the acute injection of fentanyl and fentanyl-treated water, while a control group received s.c. saline instead of 0.2 mg/kg fentanyl and drank untreated water—thus remaining opioid-naïve prior to the administration of naloxone on drinking day 8. Following the hot water drop, peak z-scored Ca²⁺ responses and 5-s AUC were significantly higher during fentanyl withdrawal compared to both baseline and acute fentanyl conditions (Figs. 3e–g and S4e–g). In contrast, acute saline or naloxone administration in opioid-naïve animals did not significantly alter the response. For the aversive airpuff, peak z-scored, but not dF/F, Ca²⁺ responses were significantly lower following acute fentanyl administration but remained unchanged during withdrawal, while for water-drinking mice peak z-score, but not dF/F, was slightly elevated after withdrawal (Figs. 3h–j and S4h–j). AUC analyses showed no significant differences in either fentanyl-treated or water-treated mice (Figs. 3j and S4j). Thus, our results indicate that CeLC^PKCδ^ neurons exhibit heightened sensitivity to noxious stimuli during fentanyl withdrawal, but reduced responsiveness to an airpuff following an acute dose of fentanyl.

### Behavior profile of spontaneous withdrawal from fentanyl drinking

We next sought to understand whether the increase in neural activity seen during fentanyl withdrawal was necessary for physical dependence. We first characterized the spontaneous withdrawal phenotype of our fentanyl drinking procedure in *Prkcd*-Cre mice 20–24 h after the removal of the fentanyl-treated water (Fig. 4a). Both male and female *Prkcd*-Cre mice displayed expected signs of opioid withdrawal, with fentanyl-drinking mice exhibiting significantly higher global withdrawal scores than water-drinking mice (Figs. 4b and S5b). Given that previous work has observed affective and somatic opioid withdrawal signs existing independently [25, 55], we categorized withdrawal behaviors into three broad categories of the signs commonly seen in rodent and human models of opioid withdrawal: shakes/tremors, autonomic signs, and affective signs (Fig. 4c–e). Fentanyl withdrawal significantly increased shakes and tremors (Figs. 4c and S5c–f) and autonomic withdrawal signs (Figs. 4d and S5g, h), but did not significantly increase affective behaviors compared to water-drinking controls (Figs. 4e and S5i–m). Additionally, fentanyl-withdrawing mice exhibited significantly reduced locomotor activity during the 15-min spontaneous withdrawal period (Fig. 4f). Since chronic opioid use can lead to paradoxical hyperalgesia [56, 57], and pain recurrence is common during opioid withdrawal in humans [57, 58], we also assessed mechanical and thermal pain sensitivity. However, we did not detect opioid-induced hyperalgesia or spontaneous withdrawal-induced hyperalgesia (Fig. S6).

### CeLC^PKCδ^ neuron inhibition does not alter pain sensitivity but prevents fentanyl withdrawal symptoms

To assess whether CeLC^PKCδ^ neuron activity is required for fentanyl dependence, we chronically inhibited these neurons using the potassium channel Kir2.1 [59–61]. We injected a Cre-dependent Kir2.1-zsGreen (AAVDJ-*CMV*-DIO-Kir2.1-zsGreen) or a control EGFP-expressing vector (AAV5-*hSyn*-DIO-EGFP) into the CeLC of *Prkcd-*Cre mice before fentanyl exposure (Figs. 4g–i and S7a). Given prior evidence that CeLC^PKCδ^ neurons contribute to nociceptive processing [12, 13, 62] and our fiber photometry results showing that these neurons respond to hot water application in opioid-naïve female mice, we first tested whether Kir2.1 overexpression affected pain sensitivity. There were no significant differences in mechanical (Fig. 4j) or thermal (Fig. 4l) withdrawal thresholds or affective-motivational responses to noxious hot water (Fig. 4k) between EGFP- and Kir2.1-expressing mice. Consistent with previous observations that inhibition of CeLC^PKCδ^ neurons impacts nociceptive responses only after injury [13], these findings indicate that CeLC^PKCδ^ neurons baseline activity is not required for mechanical or thermal nociception in opioid-naïve mice.

### CeLC^PKCδ^ neuron inhibition prevents spontaneous fentanyl withdrawal symptoms

Next, we assessed whether Kir2.1-expressing mice developed spontaneous fentanyl withdrawal. Importantly, both EGFP- and Kir2.1-expressing mice consumed comparable amounts of fentanyl (Fig. 4m). As expected, EGFP-expressing, fentanyl-drinking mice exhibited significantly higher withdrawal scores than did Kir2.1-expressing, water-drinking mice (Fig. 4n). Kir2.1 expression prevented withdrawal symptoms in fentanyl-drinking mice, however: Kir2.1-expressing, fentanyl-drinking mice did not show significantly higher withdrawal scores than water-drinking controls, and their scores were significantly lower than those of EGFP-expressing, fentanyl-drinking mice (Fig. 4n). This pattern was also observed for shakes/tremors (Fig. 4o) and autonomic withdrawal symptoms (Fig. 4p), though this effect was not statistically significant for the autonomic subscore. Additionally, affective withdrawal symptoms did not significantly differ between groups (Fig. 4q). The primary withdrawal-related behaviors that were reduced by Kir2.1 expression were resting tremors and paw tremors (Fig. S7b–l). Lastly, while fentanyl withdrawal typically reduces locomotor activity (Fig. 4f), we observed a small but nonsignificant increase in locomotion in both fentanyl-drinking groups compared to water-drinking, Kir2.1-expressing mice (Fig. 4r). This suggests that CeLC^PKCδ^ inhibition may modulate locomotor activity in the absence of fentanyl withdrawal.

### Brain-wide identification of opioid-sensitive inputs to CeLC^PKCδ^ neurons

Our findings indicate that CeLC^PKCδ^ neurons respond to both precipitated and spontaneous withdrawal from fentanyl. To determine whether these effects are mediated by opioid-sensitive inputs to CeLC^PKCδ^ neurons, we used viral-mediated circuit tracing to identify brain regions that meet two criteria: (1) provide monosynaptic input to CeLC^PKCδ^ neurons, and (2) express MORs on CeA-projecting neurons. To map monosynaptic inputs to CeLC^PKCδ^ neurons, we performed rabies-mediated retrograde tracing in *Prkcd*-Cre mice (Fig. 5a–c). We injected a mixture of Cre-dependent TVA-mCherry and G-protein “helper” viruses into the CeLC of *Prkcd-*Cre mice 2 weeks prior to the injection of EnvA-pseudotyped RABV*d*G-GFP, which then selectively enters PKCδ⁺ neurons via the TVA receptor and retrogradely “hops” only one synapse using the G-protein.

Consistent with previous studies [34], we observed dense monosynaptic inputs from the striatum, amygdala, and extended amygdala, as well as substantial projections from sensory cortical areas, including olfactory, somatosensory, and auditory regions (Fig. 5c). We also identified inputs from the thalamus, hypothalamus, and pallidum, with the parabrachial nucleus (PBN) serving as the primary hindbrain input (Fig. 5c). Notably, we did not detect labeled cells in the contralateral CeA, suggesting minimal interhemispheric connectivity within the CeLC^PKCδ^ population.

Next, we used a retrogradely trafficked, Cre-dependent color-switching viral tracer (AAVretrograde-*EF1α*-Nuc(flox)-mCherry-EGFP) in *Oprm1*-Cre mice to assess the density of MOR-expressing inputs to the CeA in the regions identified via RABV*d*G-mediated tracing. This approach labels the nuclei of *Oprm1*⁺ inputs with EGFP and *Oprm1*⁻ inputs with mCherry (Fig. 5d). The distribution of inputs in both hemispheres was consistent between RABV*d*G and color-switching tracer mice (Figs. 5e, S8 and S9). All examined brain regions contained both *Oprm1*⁺ and *Oprm1*⁻ inputs. Other amygdalar areas and sensory cortical areas showed particularly high proportions of *Oprm1*^*+*^ inputs. (Figs. 5e and S8) Both tracing strategies labeled comparatively few contralateral inputs to the CeA (Fig. S9). Finally, to determine whether CeLC^PKCδ^ neurons are part of the ascending spinoparabrachial nociceptive pathway, we further characterized parabrachial projections to these neurons. We identified MOR-expressing monosynaptic projections to PKCδ⁺ CeA neurons, indicating a potential role for this pathway in opioid drug effects (Fig. S10).

## Discussion

Neural activity within the CeA is crucial for processing negative emotional states and aversive experiences, including those linked to opioid withdrawal. Though recent research increasingly illuminates the diversity of CeA cell-types and their associated neural circuits, significant gaps remain regarding how the specific cell-types and pathways in the CeA sustain physical dependence on opioid drugs. Using cell-type-specific calcium imaging, activity modulation, and brain-wide circuit mapping, we identify CeLC^PKCδ^ neurons as critical drivers of opioid withdrawal. Collectively, our data indicate that CeLC^PKCδ^ neurons are hyperactive and hypersensitive to noxious stimuli during opioid withdrawal, that reducing their activity can alleviate withdrawal, and that several input pathways to CeLC^PKCδ^ neurons may be directly modulated by opioid drugs via MORs.

Previous studies reported CeA cellular activity during opioid withdrawal [21–25]. Our immunohistochemistry results (Fig. 1) and our photometry results (Fig. 3) extend these findings in two major ways. First, we identify CeLC^PKCδ^ neurons in the anterior half of the CeA as a highly activated population. Second, we report that the activation is both rapid and sustained. The most straightforward explanation for chronic fentanyl-induced changes in CeLC^PKCδ^ neural activity is that MORs and/or other opioid receptors are present in the circuitry that controls the activity of these neurons. Though MORs are G_i/o_-coupled receptors, chronic MOR stimulation (*e.g*., by chronic fentanyl exposure) increases adenylyl cyclase expression and cAMP-mediated signal transduction, including depolarizing mechanisms, in MOR-expressing neurons [63–67]. This paradoxical increase in signal transduction helps maintain basal signaling levels despite chronic MOR-mediated suppression of these processes. The return to basal signaling during ongoing, chronic opioid exposure may be reflected in Fig. 1, where fentanyl-drinking mice did not have a significant change in FOS expression in the CeA relative to water-drinking mice when administered saline. However, that compensatory increase in transduction and cellular activity can be “unmasked” when MOR stimulation either ends (*e.g*., spontaneous withdrawal, as in Fig. 4) or is abruptly blocked (*e.g*., precipitated withdrawal, as in Figs. 1 and 3) [52, 66, 67]. This molecular process is called cellular dependence and represents a physiological state underlying physical dependence. Adenylyl cyclase-dependent hyperactivity of MOR-expressing neurons, including on CeLC^PKCδ^ neurons themselves, can explain both the increase in FOS and the sharp rise in Ca^2+^ activity that we detected during fentanyl withdrawal in CeLC^PKCδ^ neurons. Cooper et al. [12] found that approximately 40% of MOR-lineage neurons express PKCδ protein; inversely, Chaudun et al. [25] found that ~80% of *Oprm1*+ cells in the CeA also express *Prkcd* mRNA. Thus, there is a strong possibility that a subset of CeLC^PKCδ^ neurons can be directly modulated by chronic fentanyl via expression of fentanyl’s primary molecular target.

Overexpression of the potassium channel Kir2.1 in CeLC^PKCδ^ neurons largely prevented fentanyl withdrawal, presumably by quelling the hypertrophy of particular molecular pathways associated with the allostatic state (Fig. 4). Interestingly, Kir2.1-containing viral vector’s spread was limited to modest coverage of the CeLC, indicating that fewer neurons were transfected than would be expected to be activated during withdrawal (Figs. 1 and S7a). Yet Kir2.1-mediated inhibition, even unilateral inhibition, nonetheless blocked fentanyl withdrawal from occurring, even with a unilateral injection (Fig. 4). The anatomical role of CeLC^PKCδ^ neurons as interneurons within the CeLC may help explain how this relatively limited number of cells could have such dramatic behavioral effects. CeLC^PKCδ^ neurons primarily synapse within the CeA, including onto projection neurons located in the CeM [37, 38]. Additionally, CeLC^PKCδ^ neurons make inhibitory synapses within the CeLC, including other CeLC^PKCδ^ neurons as well as PKCδ^-^ neurons. CeLC^PKCδ^ neurons thus have the ability to regulate multiple CeA cell-types and gate the activity of the structure as a whole. These varied roles may help explain how a relatively small proportion of CeLC neurons, when silenced long-term, might perturb a much larger network to produce robust behavioral results such as those seen here.

An alternative explanation for the heightened activity seen here is that MOR-expressing, and therefore opioid-sensitive, excitatory projections to the CeA are susceptible to cellular dependence. Their activity, in turn, could drive the activity of downstream CeLC^PKCδ^ neurons. Our parallel viral labeling strategies revealed that most brain areas with direct inputs to CeLC^PKCδ^ neurons contain MOR-expressing CeA projections (Fig. 5). The projections of several of these inputs are well-established mediators of drug dependence and withdrawal, including those from the insular cortex [68–70], dorsal striatum [71], basolateral amygdala [72, 73], and paraventricular thalamus [74, 75]. Cellular dependence within these input neurons provides a potential presynaptic mechanism for the hyperexcitability seen in the CeA during withdrawal.

While many of the areas we identified in our tracing studies are well-established loci of the effects of opioid drugs, the role of sensory cortical areas in opioid dependence may be underexplored. Our photometry results in naïve mice suggest that the immediate CeLC^PKCδ^ response to stimuli relates more to stimulus content than behavioral response (Figs. 2 and S3). This finding suggests that the activity we report might be driven, at least in part, by direct sensory input pathways. Furthermore, we found that both acute fentanyl administration and fentanyl withdrawal modulate the response of CeLC^PKCδ^ neurons to somatosensory stimuli (noxious hot water, airpuff; Fig. 3). Airpuff also has an auditory component, as the release of compressed air is accompanied by a loud puffing sound. These results further suggest that opioids might affect the inputs from areas of the brain responsible for processing these two sensations. Several areas our tracing approach identified participate in somatosensation and audition, including the somatosensory cortex, the secondary auditory cortex, and the temporal association area, as well as the ventral posterior thalamic nucleus, part of the thalamic auditory system (Figs. 5 and S8). In further support of sensory areas as sites of action of opioid drugs, emerging evidence shows a susceptibility of the mouse somatosensory and auditory cortex to persistent plasticity after perinatal opioid exposure [76, 77]. It is unknown whether such findings translate to animals exposed as adults, or whether *Oprm1*+ projections to the central amygdala are likewise affected. Alongside our results, the susceptibility of these sensory areas to perinatal opioid exposure warrants further investigations of *Oprm1*+ CeA projections from such regions in the context of opioid dependence.

We note that molecularly defined neural populations in the central amygdala are not always homogeneous. For example, we found that the peak of *withdrawal*FOS occurs in the anterior half of the CeA, and that *withdrawal*FOS is elevated throughout the CeLC (Fig. 1e–g). Investigations of chronic pain-induced phosphorylated ERK and FOS, on the other hand, find that they increase in CeLC^PKCδ^ neurons as well, but primarily in the posterior half of the CeA [13]. Additionally, previous studies suggest that the CeA is a lateralized structure. Under this framework, the right CeA putatively dominates the processing and expression of aversion, and especially nociception [11, 15, 50, 51, 78]. In contrast, we did not detect a robust effect of hemisphere in our studies, despite withdrawal causing a highly aversive state (Fig. S2). Our findings are in agreement with other non-chronic pain states, including itch [79], latent pain sensitization [12], and isoflurane administration [80]. Taken together, these findings suggest that there are multiple CeLC^PKCδ^ functional ensembles responding to aversive stimuli that may undergo plasticity under different conditions [10, 12, 13]. Indeed, Kim et al. [33] suggested that functional separation of PKCδ^+^ neurons exists along the anatomical axes of the CeA. Specifically, they found that CeC^PKCδ^ neurons in the anterior CeA receive different intra-amygdalar inputs, respond differently to aversive stimuli, and differentially drive behavior compared to CeL^PKCδ^ neurons located more posterior. Spatial transcriptomic analyses also show genetic divisions of CeLC^PKCδ^ neurons along the anatomical axes of the CeA [26, 27]. Here, we determined that *withdrawal*FOS peaks in the anterior-to-middle portion of the CeA, but is present throughout the anterior-posterior axis of the CeA (Fig. 1), suggesting overlap with both populations identified by Kim et al. Our viral injections targeted both CeC^PKCδ^ and CeL^PKCδ^ neurons in the anterior half of the CeA, centered around the peak of *withdrawal*FOS (Figs. 1g, S3a and S7a), so may have captured some of each “distinct” population. Thus, our results might mask some of their divergent roles. Future work could investigate the possibility of multiple or overlapping functional ensembles using dual color single-cell calcium imaging approaches or activity-dependent genetic capture (e.g., *Fos*TRAP) [81].

Our studies presented here have several limitations. First, we selected a 24-h *ad libitum*, homecage, forced-access model to render our mice fentanyl-dependent. We chose this model to reduce confounding variables such as stress associated with experimenter handling and noxious drug injections that could also alter the same CeA cell-types and circuits involved in opioid withdrawal. We also wanted to avoid surgical injuries associated with implanting catheters for intravenous self-administration, which themselves can induce neuroplasticity in the central amygdala [82]. One drawback to this drinking model, however, is that it requires individual housing, an additional possible source of stress. Long-term social isolation stress is associated with neuroplastic changes within the CeA and within the BLA, which projects to CeLC^PKCδ^ neurons [83–85]. This is especially true when isolation occurs during adolescence, which somewhat limits the future utility of the model. Additionally, mice did not develop a preference for fentanyl-treated water in an identical assay where they had the choice between fentanyl- and untreated water (Fig. S1f–h). This limits our ability to draw conclusions about drug-seeking behavior from these studies. Finally, we lack statistical power to detect sex differences in our photometry Ca^2+^ imaging experiments (Fig. 3). While we did not detect profound sex effects in these studies, we did find that the area under the curve of the Ca^2+^ response to noxious stimuli showed differences between male and female subjects (Fig. 2). Given evidence of sexual dimorphism in CeA genetic systems and functional responses (e.g., CGRP signaling in pain [86, 87] and responses to alcohol [88]), future work should evaluate whether PKCδ⁺ CeLC neurons are differentially activated by sex during opioid withdrawal.

The aversive experience of opioid withdrawal makes abstinence challenging and increases the risk of relapse. The high comorbidity between persistent pain and OUD suggests shared biological mechanisms, and our findings, which align with a model of latent pain sensitization, indicate that CeLC^PKCδ^ hyperactivity may be one such mechanism. By identifying the role of the nociception-activated and -responsive CeLC^PKCδ^ neurons in opioid withdrawal, our study reveals how a neural circuit involved in pain processing is affected by chronic opioid use. In total, these results underscore the need for cell-type-specific investigations within the CeA, which will be essential for developing targeted treatments for OUD that do not compromise pain management.

## Supplementary information

Wooldridge et al Supplementary materials

## Data Availability

All data are available in the main text or the Supplementary Materials.
