# Supplementary material for: Central amygdalar PKCδ neurons mediate fentanyl withdrawal
Source: Neuropsychopharmacology. 2025 Dec 19;51(6):1110–22. doi: 10.1038/s41386-025-02298-7 (PMC13125285; doi:10.1038/s41386-025-02298-7)
Supplement: Supplementary file 1 — Wooldridge et al Supplementary materials [file 41386_2025_2298_MOESM1_ESM.docx]

Supplementary Materials for

Central amygdalar PKCδ neurons mediate fentanyl withdrawal

Lisa M. Wooldridge *et al.*

*Correspondence to: Gregory Corder.

Email: gcorder@upenn.edu

**This PDF file includes:**

Figures S1-10

Supplementary Materials and Methods

Supplementary Results

Supplement References

**
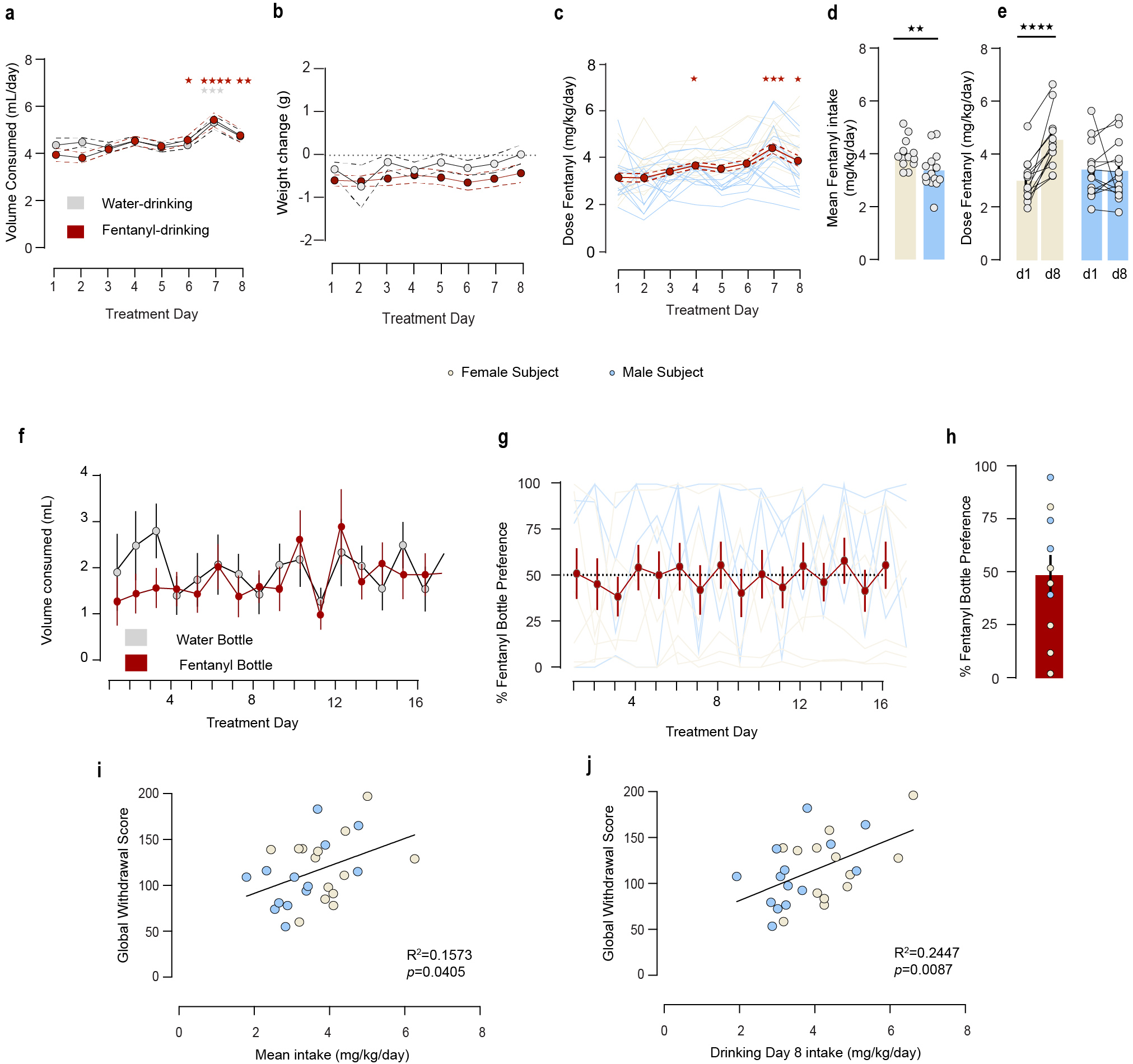
Figure S1. Characterization of fentanyl drinking model.** (**a**)-(**e**) 24hr home-cage, ad libitum access to either untreated (water-drinking, n=12F/13M) or treated with 0.02 mg/mL fentanyl (n=13F/14M). **(a)** Both fentanyl-drinking (red) and water-drinking (grey) mice drank significantly more on treatment day 7, and fentanyl-drinking mice drank significantly more on days 6 and 8, compared to treatment day 1. Two-way repeated measures ANOVA with Bonferroni’s correction, significant effect of treatment day (F(7, 350)=13.02, *p*<0.0001) and subject (F(30, 350)=9.515, *p*<0.0001). **p*=0.0405, ***p*=0.0019, ****p*=0.0004, *****p*<0.0001. Points represent average +/- SEM. **(b)** No significant difference in weights across the experiment or between water-drinking and fentanyl-drinking mice (Mixed-effects analysis). **(c)** Fentanyl-drinking mice consumed significantly more fentanyl on treatment days 4, 7 and 8 compared to treatment day 1 (One-way repeated measures ANOVA, significant effect of treatment (F(4.015, 104.4)=12.11, *p*<0.0001) and subject (F(26, 182)=10.45, *p*<0.0001)). Points represent the mean +/- SEM dose consumed on each treatment day; lines represent individual mice’s trajectories (tan: female mice; blue: male mice). **p*<0.05, ****p*=0.0001 **(d)** Females drank significantly more fentanyl than male mice (Unpaired t-test, t=2.446, **p=*0.0218*).* **(e)** Females, but not males, drank significantly more fentanyl on experimental day 8 compared to day 1 (Two-way repeated measures ANOVA, significant effect of experimental day (F(1, 50)=7.428, *p*=0.0088) and sex x experimental day interaction (F(1,50)=8.446, *p*=0.0054). ****p*=0.0006 **(f)-(h)** Fentanyl drinking model does not induce a fentanyl preference in a two-bottle choice assay (n=6F, 4M). **(f)** Mice consumed similar volumes from the water and fentanyl bottle on every treatment day (Mixed-effects analysis). Points represent the mean +/- SEM volume consumed from each bottle on each day. **(g)** The preference for the fentanyl bottle vs. the water bottle did not increase over the course of the two-bottle choice paradigm (Mixed effects analysis) Points represent the mean +/- SEM dose consumed on each treatment day; lines represent individual mice’s trajectories (tan: female mice; blue: male mice). **(h)** The mean preference scores for individual animals ranged from 2.32-94.88% preference for the fentanyl bottle over the water bottle. Bar represents mean % fentanyl bottle preference +/- SEM. **(i)-(j)** Global withdrawal scores directly correlate to the **(i)** mean dose fentanyl consumed across the 8 treatment days (Simple linear regression) and **(j)** dose consumed on treatment day 8 (Simple linear regression). Water-drinking: n=12F/13M; fentanyl-drinking: n=13F/14M


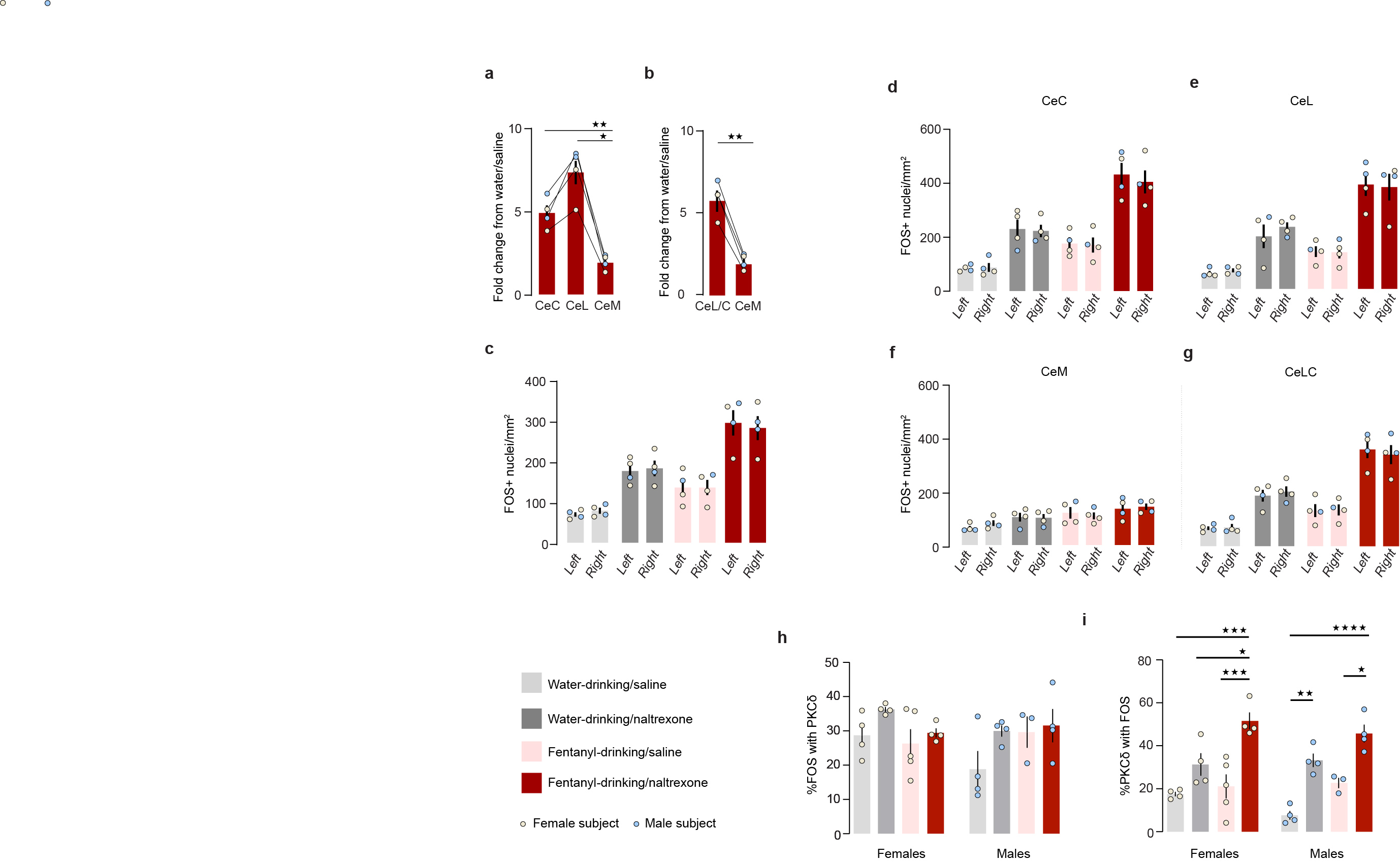


**Figure S2. Further characterization of FOS+ nuclei in the CeA during fentanyl withdrawal.** **(a)** Normalized fold-change in FOS+ nuclei in the fentanyl-drinking/naltrexone group, normalized to water-drinking/saline condition. The magnitude of the change was greater in the CeC and CeL than in the CeM. One-way Repeated measures ANOVA with Bonferroni’s correction, significant effect of subregion (F(1.272, 3.81)=59.94; *p*=0.0016) and subject (F(3,9)=10.52, *p*=0.0027).**p*=0.0102, ***p*=0.0042. Bars represent mean +/- SEM values; dots represent individual subject values (tan=females, blue=males). n=2F/2M **(b)** When considering the CeLC as a whole, we detected a greater increase in the number of FOS+ neurons in the CeLC compared to the increase in the CeM (Paired t-test, t=11.05, **p=0.0016). **(c)** No significant effect of hemisphere on the expression of FOS in the CeA as a whole (Three-way repeated measures ANOVA, main effect of drinking condition (F(1,12)=21.01, p=0.0006), and naltrexone condition (F(1,12)=48.06, *p*<0.0001) **(d)** No main effect of hemisphere on the numbers of FOS+ nuclei in the CeC (Three-way repeated measures ANOVA, main effects of drinking condition (F(1,12)=28.20, p=0.0002), naltrexone condition (F(1,24)=53.77, p<0.0001) **(e)** No main effect of hemisphere on the numbers of FOS+ nuclei in the CeL (Three-way repeated measures ANOVA, main effects of drinking condition (F(1,12)=22.72, *p*=0.0005), naltrexone condition (F(1,24)=59.69, *p*<0.0001). **(f)** A significant side x drinking condition x naltrexone condition interaction was detected in the CeM (Three-way repeated measures ANOVA, significant effect of drinking condition (F(1,12)=7.431, *p*=0.0184), side x drinking x naltrexone interaction (F(1,12)=5.598, *p*=0.0357), but no comparisons were signicant after correcting for multiple comparisons. **(g)** No main effect of hemisphere on the numbers of FOS+ nuclei in the CeLC as a whole (Three-way repeated measures ANOVA, main effect of drinking condition (F(1,12)=28.30, p=0.0002), naltrexone (F(1,12)=68.90, p<0.0001), and drinking x naltrexone interaction (F(1,12)=4.763, p=0.0497). (**h**) A three-way ANOVA detected a sex x drinking treatment interaction on the % of FOS+ cells containing PKCdelta immunoreactivity, (main effect of withdrawal condition (F(1,24)=5.253, *p*=0.0310), sex x drinking condition interaction (F(1,24)=4.357, *p*=0.0477)) but no comparisons were significant after correcting for multiple comparisons (Bonferroni’s correction). Water-drinking/saline: n=4F/4M; water-drinking/naltrexone: n=4F/4M; fentanyl-drinking/saline: n=5F/3M; fentanyl-drinking/naltrexone: n=4F/4M (**i**) A three-way ANOVA detected no significant effect of sex (main effect of drinking (F(1,24)=20, p=0.0002), naltrexone condition (F(1,24)=65.43, p<0.0001). **p<*0.05, ***p<*0.01, ****p<*0.001, *****p*<0.0001

**
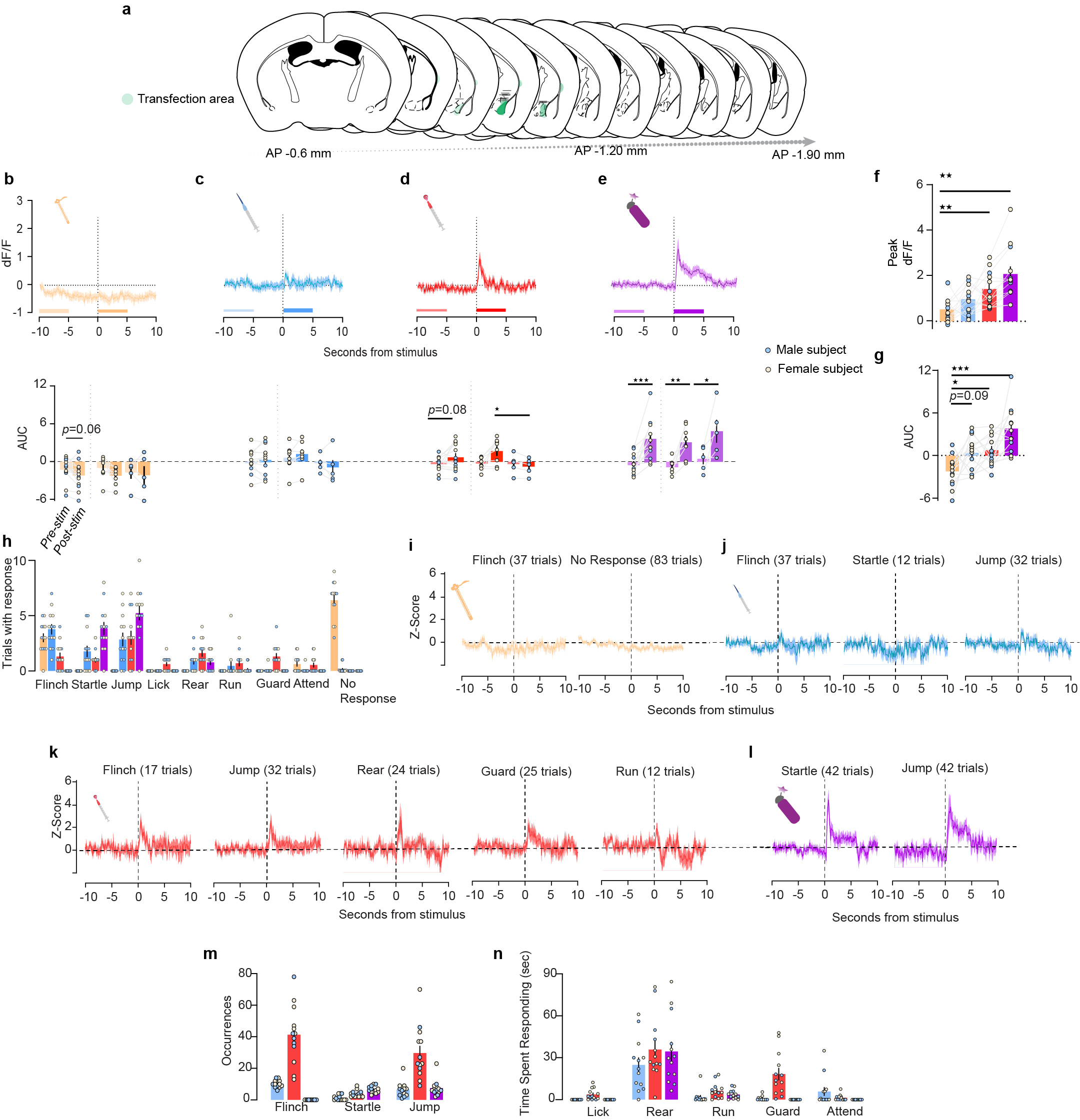
**

**Figure S3. CeLC^PKCδ^ neurons in opioid-naïve mice respond to noxious aversive and non-noxious aversive stimuli. (a)** Approximate location of fiber tip (lines), and viral expression on the same slice (green area), for each subject. n=8F/5M **(b)-(e)** Top: peri-stimulus time histogram of non-normalized dF/F from -10sec to 10sec from the moment of stimulus application, following application of **(b)** a 0.16g von Frey filament (innocuous light touch); **(c)** noxious pinprick with a 25G needle (noxious pinprick); **(d)** a 55°C hot water drop (noxious hot water); and **(e)** an aversive, but non-noxious, airpuff delivered to the side of the face contralateral to virus injection and fiber. Lines and area fill represent mean dF/F of 10 trials/subject, averaged across all subjects, +/- SEM. Bottom: area under the dF/F curve (A.U.C) prior to (-10 sec to -5 sec) vs. after (0 sec to 5 sec) each stimulus. Left: All animals, middle: females, right: males. **(b)** Compared to pre-stimulus baseline, innocuous light touch was associated with a decrease in the AUC, but this observation did not reach significance (Paired t-test, t=2.068). No significant effects of sex (Repeated Measures Two-way ANOVA, significant effect of subject only (F(11,11)=5.518, *p*=0.0043. Bars represent mean dF/F +/- SEM; dots represent individual points. **(c)** Noxious pinprick did not significantly affect area under the dF/F curve (Paired t-test); there was no significant effects of sex on area under the dF/F curve (Two-way repeated measures ANOVA) **(d)** Noxious hot water did not significantly affect area under the dF/F curve (Paired t-test); Two-way repeated measures ANOVA detected a significant Sex x Timepoint interaction (F(1,11)=5.72, *p=*0.0357), where females had higher AUCs than males at the post-stimulus timepoint (Bonferroni’s correction; *p*=0.0203) **(e)** Aversive airpuff was associated with a significant increase in area under the dF/F curve (Unpaired t-test, t=4.708, ****p*=0.0005) in both male and female mice (Two-way repeated measures ANOVA, significant effect of timepoint (F(1, 11) =19.83, *p*=0.001. **p*=0.0123, ***p=*0.006 **(f)** Compared to innocuous light touch, aversive airpuff and hot water produced a significant increase in the peak dF/F (One-way repeated measures ANOVA, (F(2.050, 24.60)=30.32, *p*<0.0001; significant effect of subject, F(12, 36)=11.20, *p*<0.0001)). ***p*<0.01 **(k)** Compared to innocuous light touch, aversive airpuff and hot water produced a significant increase in the AUC (One-way ANOVA with repeated measures and Bonferroni’s correction; significant effect of treatment (F(2.580, 30.96)=11.35, p<0.0001); **p*=0.0114, ***p=0.0009; pinprick AUC was slightly raised, but it did not reach statistical significance (*p*=0.091) **(h)** Summary of immediate behavioral response to stimuli. Bars represent mean # of trials with a given response across all animals; points represent individual subjects. Female subjects: tan dots; male subjects: blue dots. Yellow: innocuous touch; blue: noxious pinprick; red: noxious hot water; purple: aversive airpuff **(j)** z-scored PETHS for (i) innocuous touch, **(j)** pinprick, **(k)** hot water, or **(l)** airpuff trials resulting in the indicated behavior. Lines and area fill indicates average +/- SEM of number of trials indicated. (**m**)-(**n**) Total occurrences (**m**) or duration (**n**) of individual behavioral responses in the 30 seconds following stimulus application, across all trials (max duration: 100 seconds). All behavioral responses that were observed are indicated. Bars represent mean occurrence # or time across all animals; individual points represent total occurrence number or time responding across all trials for a given subject (females: tan dots, males: blue dots). Because few trials resulted in more than a flinch, innocuous touch behaviors were only quantified at the initial timepoint and not for 30seconds following.


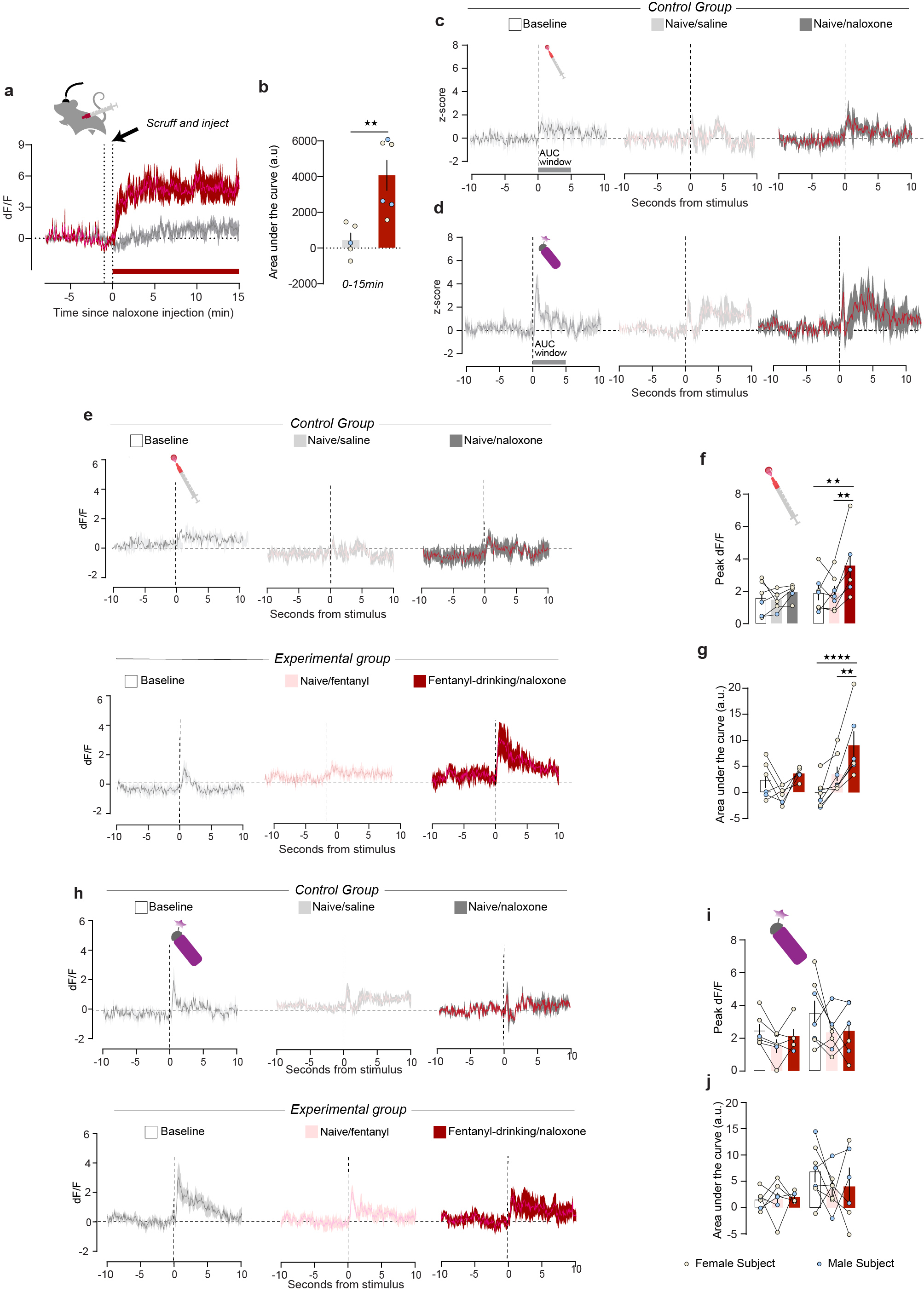


**Figure S4. CeLC^PKCδ^ neurons are hyperactive and hypersensitive during fentanyl withdrawal. (a)** Peri-injection time histogram of fluorescence before, during, and after an injection of 3 mg/kg naloxone in control, opioid-naïve animals (dark gray) and fentanyl-dependent animals (red). Red bar: AUC interval in **(b)**. Lines and area fills represent mean +/- SEM of the dF/F fluorescence for each timepoint; data were collected at 10Hz and downsampled to 3Hz during postprocessing. Water-drinking/naloxone: n=4F/1M; Fentanyl-drinking/naloxone: n=3F/3M **(b)** The net AUC for the 15 min after naloxone injection is significantly higher for fentanyl-drinking vs. naïve mice (Unpaired t-test, t=3.631, ***p*=0.0055). **(c)-(d)** Peri-stimulus time histogram of the z-scored fluorescence from 10 sec prior to 10sec following application of a noxious hot water drop **(c)** or airpuff **(d)** for the water-drinking group on the baseline stimulus test day (left), after an injection of saline (middle), or after an injection of naloxone (right). Lines and area fill represent mean values of five trials averaged across subjects. Water-drinking: n=4F/2M for baseline and acute fentanyl and n=4F/1M for withdrawal. Fentanyl-drinking: n=4F/3M for baseline and acute fentanyl and 3F/3M for withdrawal. **(e)** Peri-event time histogram of the dF/F fluorescence response to noxious hot water for the water drinking group (top) and fentanyl-drinking group (bottom) following each treatment condition. **(f)** Peak dF/F significantly increased following the application of a noxious hot water drop during withdrawal compared to the same animals at baseline or following 0.2 mg/kg fentanyl (Mixed-effects analysis with repeated measures and Bonferroni’s correction; significant effect of Timepoint (F(2,20)=7.215, *p*=0.0044). ***p*=0.0018 (Baseline vs. fentanyl/naloxone) or *p*=0.0021 (Acute fentanyl vs. fentanyl/naloxone). **(g)** The AUC of the dF/F curve for noxious hot water response curve was significantly higher during fentanyl withdrawal than at baseline (Mixed-effects analysis with repeated measures and Bonferroni’s correction, significant effect of timepoint (F(2,20)=12.18, *p*=0.0003), Treatment x Timepoint interaction (F(2,20)=6.926, *p*=0.0052)) ***p*=0.0042, *****p<*0.0001 **(h)** Peri-event time histogram of the dF/F fluorescence response to airpuff for the water drinking group (top) and fentanyl-drinking group (bottom) following each treatment condition. **(i)** There was no effect of treatment group or experimental timepoint on the peak dF/F fluorescence response to an aversive airpuff (Mixed-effects analysis with repeated measures). **(j)** There was no effect of treatment group or experimental timepoint on the AUC of the dF/F curve an aversive airpuff (Mixed-effects analysis with repeated measures)

**
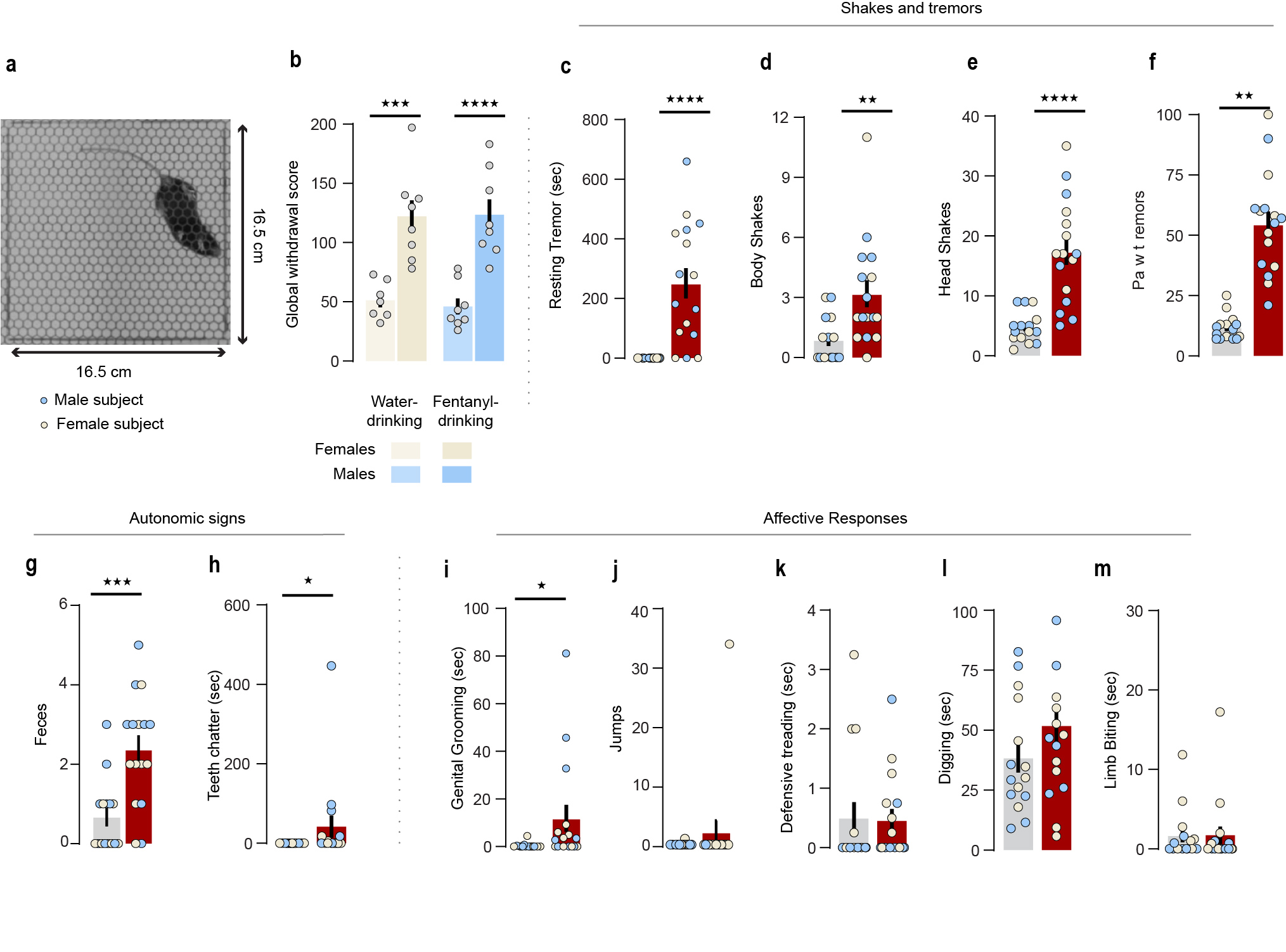
Figure S5. Further characterization of fentanyl dependence phenotype.**

**(a)** View of mouse during withdrawal scoring. Cameras mounted directly underneath the observation chamber were placed inside a Plexiglas box, allowed for a clear view of the animal’s jaw and paws. Videos were recorded under red light and desaturated to facilitate scoring. (**b)** Global withdrawal scores were similar between water-drinking male and female mice (light bars) and between fentanyl-drinking male and female mice (dark bars; Two-way ANOVA, main effect of treatment (F(1,27)=48.10, p<0.0001; ***p=0.0002, ****p<0.0001). Male subjects represented as blue bars or dots; female subjects represented as tan bars or dots. Water-drinking: 7F/8M, Fentanyl-drinking: 8F/8M **(c)-(m)** Individual measures that contribute to global withdrawal score. **(c)** Fentanyl-drinking mice spent significantly more time in a resting tremor vs. water-drinking mice (Mann Whitney test, U=22.50, ****p<0.0001). Purple points: female mice; green points: male mice **(d)** Fentanyl-drinking mice exhibited significantly more body shakes vs. water-drinking mice (Mann Whitney test, U=43.50, **p<0.0014). **(e)** Fentanyl-drinking mice exhibited significantly more head shakes vs. water-drinking mice (Unpaired t-test, t=5.386, ****p<0.0001). **(f)** Fentanyl-drinking mice exhibited significantly more paw tremors vs. water-drinking mice (Unpaired t-test, t=3.096, **p=0.0043). **(g)** Fentanyl withdrawal was associated with increased feces expelled compared to water-drinking mice (Unpaired t-test, t=3.994, ***p=0.0004). **(h)** Fentanyl-drinking mice exhibited significantly more teeth chattering vs. water-drinking mice (Mann Whitney test, U=75, *p=0.0177). **(i)** Fentanyl withdrawal increased time spent engaging in genital grooming (Mann Whitney test, U=66, *p=0.0143) However, fentanyl withdrawal was not associated with an increase in **(j)** jumps (Mann Whitney Test) or **(k)** time spent defensive treading (Mann Whitney test), **(l)** Digging (Unpaired t-test), or **(m)** Limb biting (Mann Whitney test).


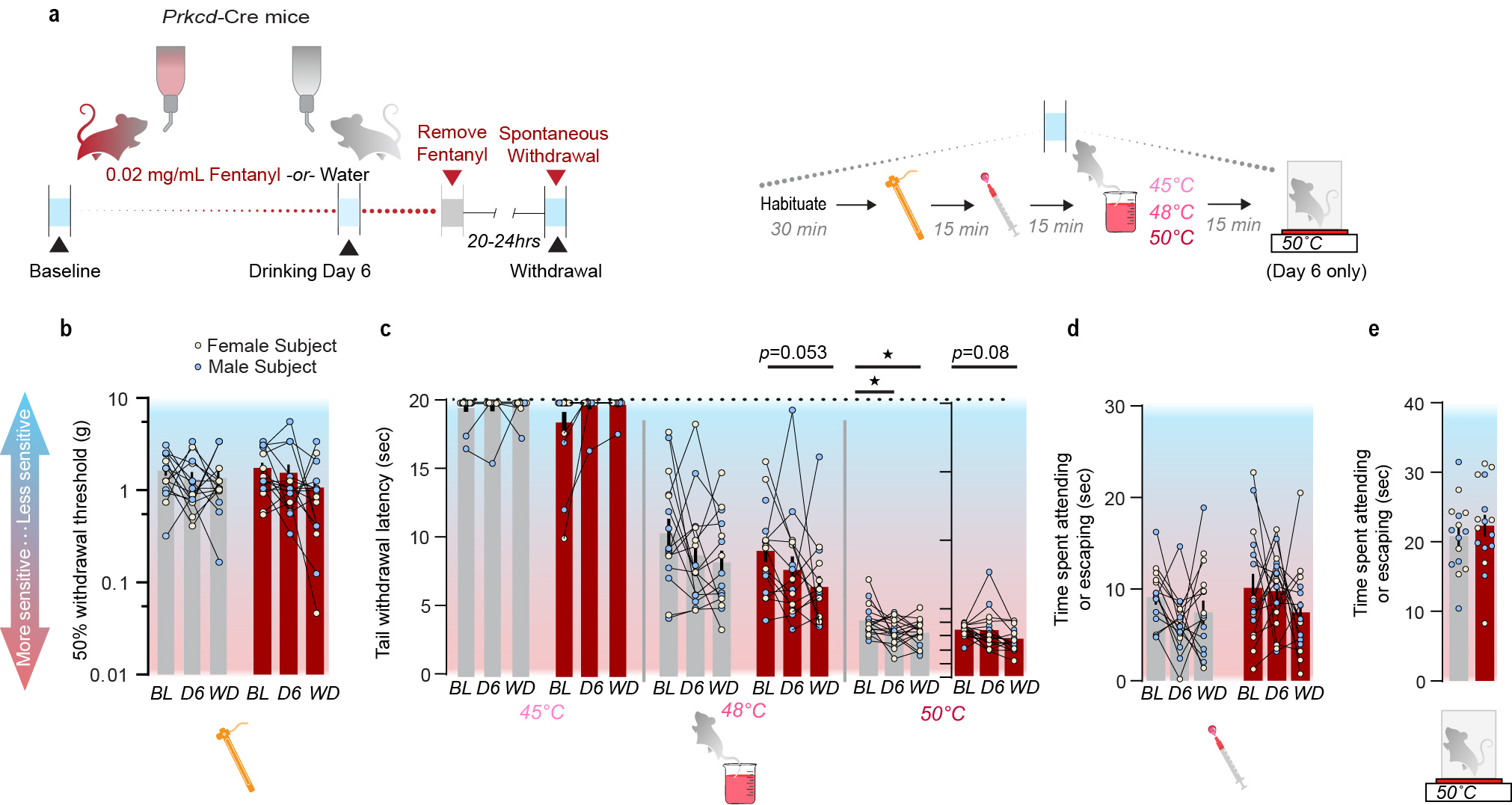

**Figure S6. Fentanyl exposure in home-cage water supply does not induce hyperalgesia during the course of treatment or during spontaneous withdrawal. (a)** Left: Experimental timeline. Test days are indicated with grey bars and black arrows. Right: outline of procedure on testing days. Because mice rapidly learn to engage in escape behaviors when repeatedly placed on an inescapable hotplate, we only tested mice on the hotplate on Treatment Day 6. Water-drinking: n=7F/8M, Fentanyl-drinking: n=8F/8M **(b)-(c)** No effect of fentanyl treatment on reflexive responses to tactile or thermal stimuli. **(b)** Six days of fentanyl treatment and spontaneous withdrawal had no effect on 50% withdrawal threshold in the von Frey Up-Down assay (Two-way Repeated Measures ANOVA). Grey bars: water-drinking mice; red bars: fentanyl-drinking mice. Bars represent mean values +/-SEM; points represent individual subjects’ data. Blue shading indicates less sensitivity to stimuli (i.e., higher nociceptive thresholds); red shading indicates more sensitivity (i.e., lower nociceptive thresholds). Female subjects: tan dots; male subjects: blue dots. (**c)** Fentanyl had no effect on mice’s latency to withdraw their tails from 45°C (left), 48°C (middle), or 50°C water (right). 45°C: Two-way repeated measures ANOVA, significant effect of subject only (F(29, 58)=2.190, p=0.0056). 48°C: Two-way repeated measures ANOVA with Bonferroni’s correction, main effect of timepoint (F(2,58)=4.185, p=0.02) and subject (F(29,58)=1.674, p=0.0476). 50°C: Two-way repeated measures ANOVA with Bonferroni’s correction, main effect of timepoint (F(2, 58)=5.520, p=0.0064). We detected no main effect of treatment group or an interaction on tail withdrawal latency at any temperature. *p<0.05. **(d)-(e)** No effect of fentanyl treatment on affective responses to noxious thermal stimuli. **(d)** No effect of fentanyl or experimental timepoint on the time spent engaging in attending or escape responses to a noxious hot water droplet applied to the left hindpaw. (Two-way repeated measures ANOVA.) **(e)** No effect of fentanyl treatment on the time spent engaging in attending or escape behaviors on an inescapable 50°C hotplate. (Two-way repeated measures ANOVA).


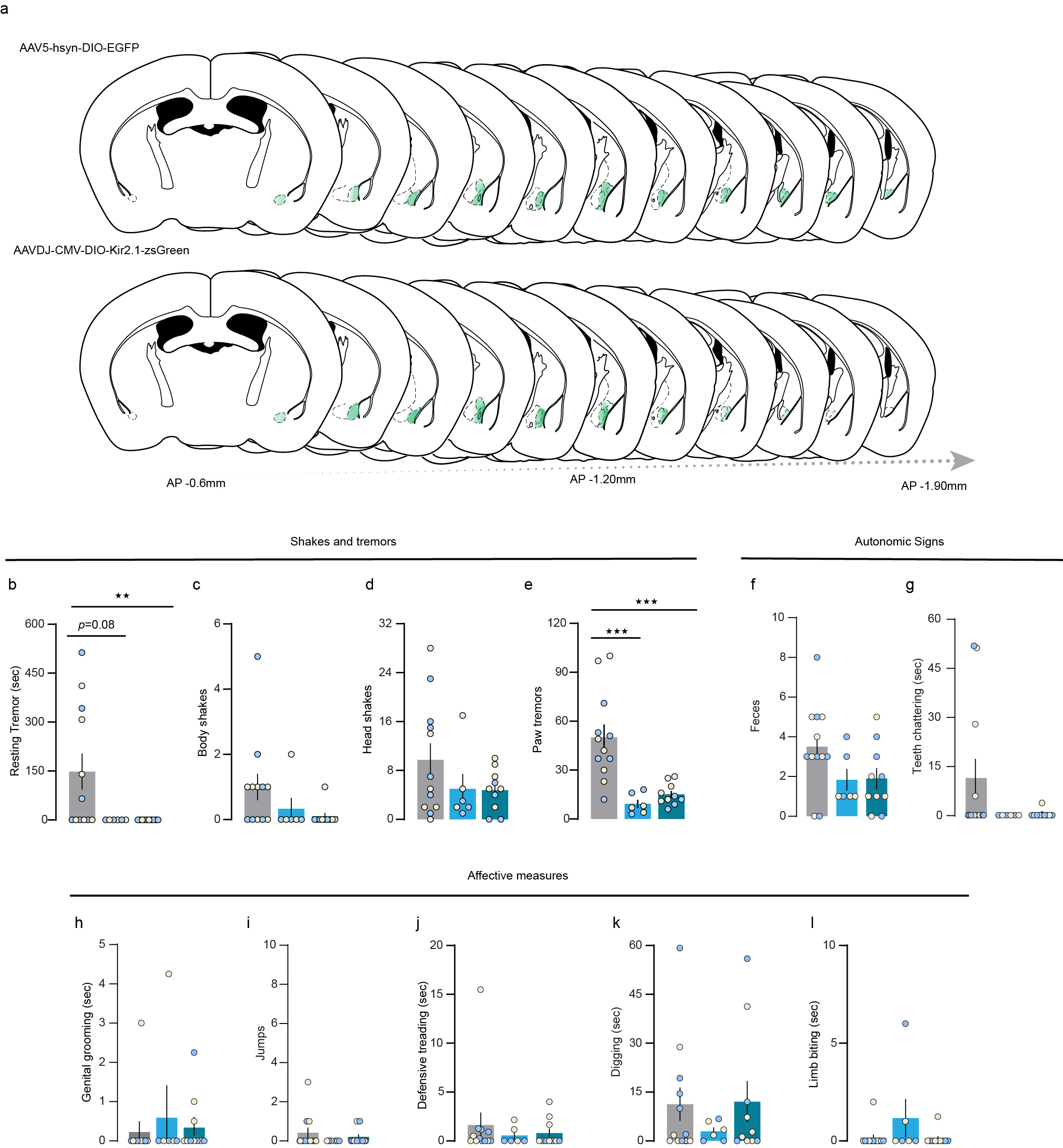


**Figure S7.** **Further characterization of Kir2.1 overexpression-suppressed withdrawal phenotype.** **(a)** Viral spread for individual subjects. Top: AAV5-hsyn-DIO-EGFP viral spread (6F/6M) Bottom: AAVDJ-CMV-DIO-Kir2.1-zsGreen viral spread for both groups of Kir2.1-overexpressing mice (9F/7M). **(b)** Kir2.1 overexpression significantly decreased the time with a resting tremor in fentanyl-dependent mice (One-way ANOVA, F(2,25) =4.666, p=0.019, *p=0.0347). Tan dots: female subjects; blue dots: male subjects. EGFP/Fentanyl: 6F/6M; Kir2.1/Water: 3F/3M; Kir2.1/Fentanyl: 6F/4M. Bars represent mean +/- SEM. Kir2.1 overexpression did not result in fewer **(c)** body shakes or **(d)** head shakes in fentanyl-drinking mice (One-way ANOVA). **(e)** Kir2.1 overexpression significantly decreased the number of paw shakes in fentanyl-dependent mice (F(2,25)=13.79, p<0.0001, ***p=0.0006). Kir2.1 overexpression did not significantly alter **(f)** the number of feces expelled, **(g)** time with teeth chattering, **(h)** time engaged in genital grooming, **(i)** jumps, **(j)** time spent defensive treading, **(k)** time spent digging, or **(l)** limb biting (One-way ANOVAs).

**
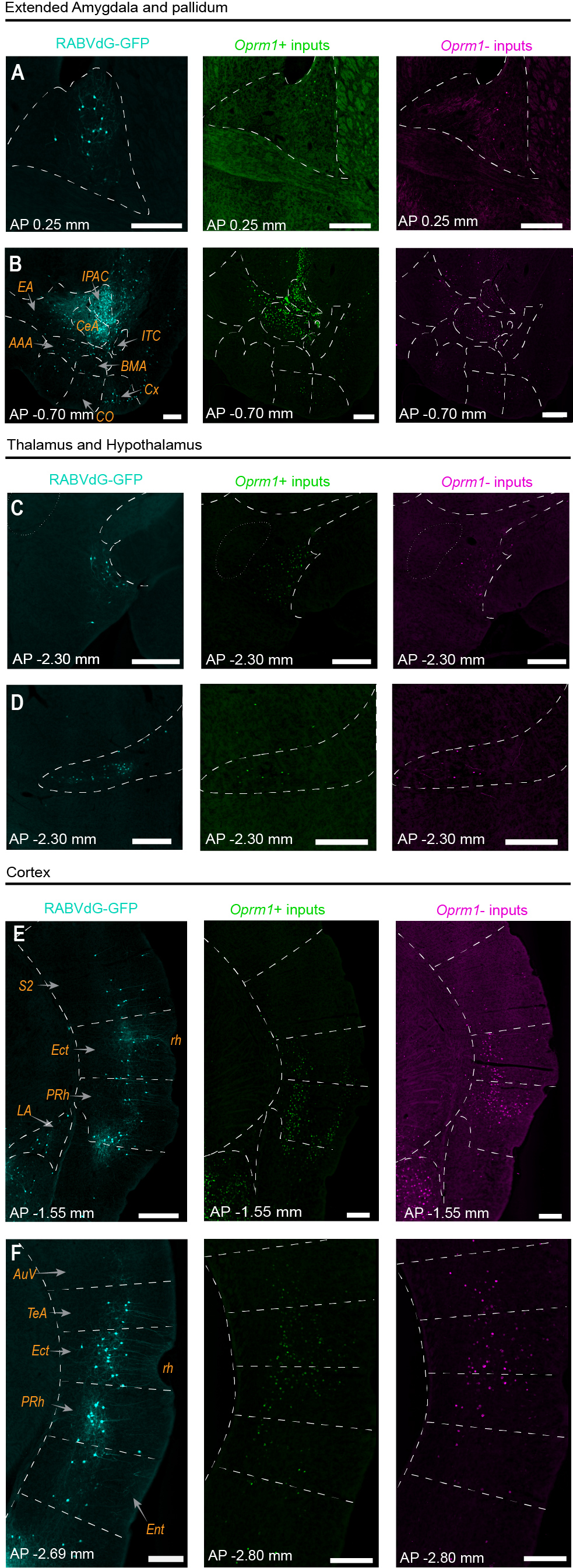
**

**Figure S8. Opioid-sensitive neurons in brain regions containing putative monosynaptic inputs to CeLC^PKCδ^ neurons. (a)**-**(b)** Representative images of RABV*d*G (cyan), EGFP (i.e., *Oprm1*+ cells; green) or mCherry (i.e., *Oprm1*- cells; magenta) labeling in the anterior extended amygdala and nearby structures. Reported values are percent *Oprm1+* cells and sum of all counted cells in that region across all mice (RABV*d*G: n=2F/2M; Flipper virus: n=3M). **(a)** Labeling in the bed nucleus of the stria terminalis (BNST), particularly the oval nucleus. 73% (1472 cells). Scale bars: 250 um. **(b)** Labeling in the anterior amygdala, extended amygdala, olfactory, and nearby regions. Scale bars: 250 um. Abbreviations: AAA: anterior amygdaloid area (60%, 1622 cells); BMA: basomedial amygdala (81%, 6262 cells); CeA: Central Amygdala CO: Cortical amygdala (71%, 8010 cells); CxA: Cortex-amygdala transition zone (70%, 1407 cells); EA: extension of the amygdala (67%, 1171 cells); END: endopiriform nucleus (75%, 3559 cells); ITC: intercalated nuclei of the amygdala (71%, 1222 cells). **(c)-(d):** Labeling in the thalamus and hypothalamus. **(c)**: labeling in the lateral hypothalamus (LH; 71%, 1902 cells), particularly in the parasubthalamic nucleus. Scale bars: 250µm. **(d)** Labeling in the ventral posterior nucleus of the thalamus (VENT; 72%, 169 cells). Scale bars: 250 um. **(e)-(f):** Labeling in the cortex. **(e)** Labeling in the medial portions of the temporal cortex. Abbreviations: ECT: ectorhinal cortex (72%, 7204 cells); PRh: perirhinal cortex (61%, 3752 cells); rh: rhinal fissure; S2: secondary somatosensory area (73%, 4792 cells)), LA: Lateral amygdala (77%, 4266 cells). Scale bars: 250um. **(f)** Labeling in the posterior temporal cortex. Abbreviations: AuV: ventral secondary auditory cortex (73%, 2024 cells); ENT: entorhinal cortex (79%, 9811 cells); TeA: Temporal association cortex (79%, 5197 cells). Scale bars: 250um.

**
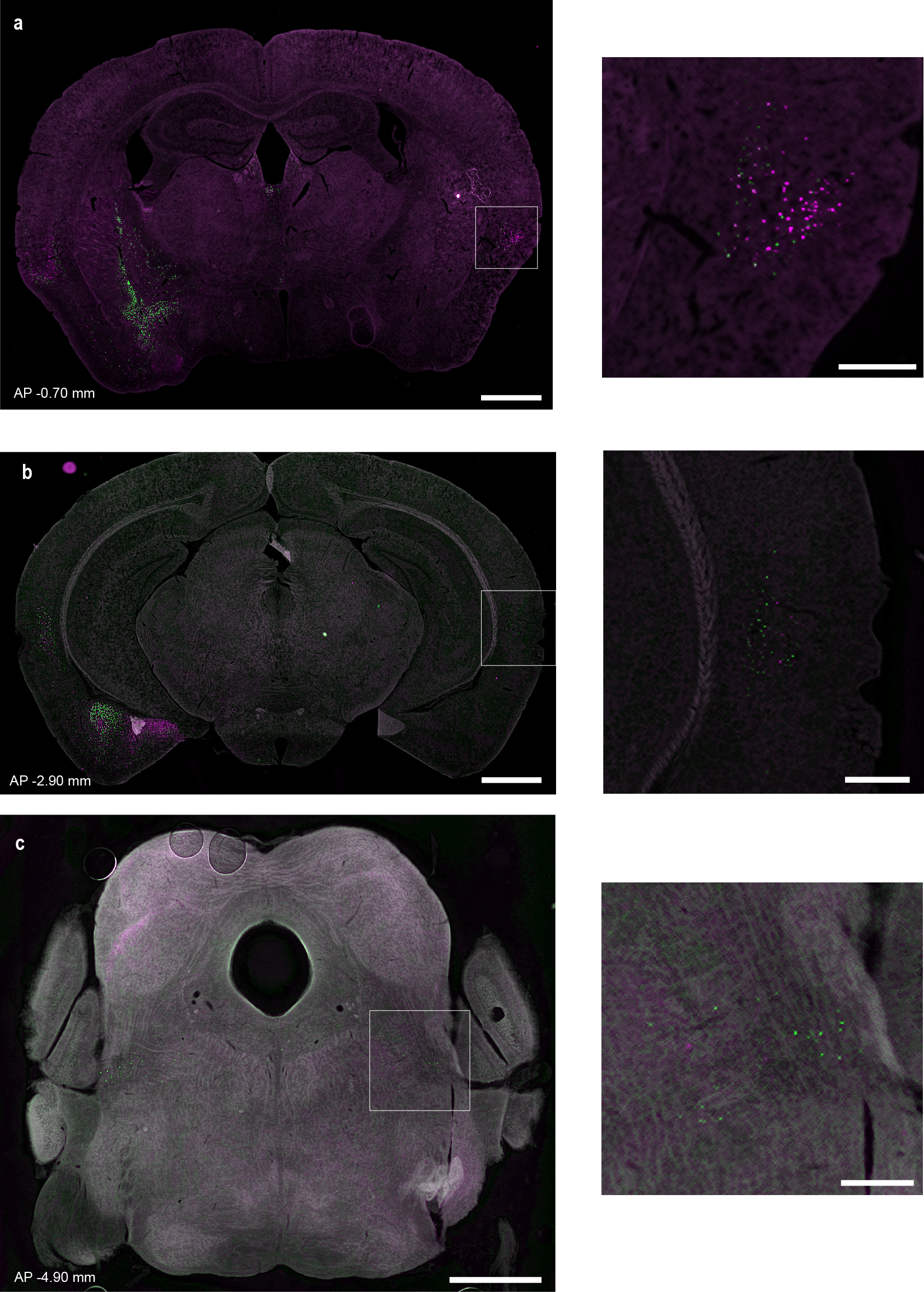
**

**Figure S9. Sparse labeling of contralateral *Oprm1*+ and *Oprm1*- inputs to the CeA with a retrograde, *Oprm1*+/*Oprm1*- neuron-labeling virus.** **(a)** Labeled nuclei are visible bilaterally in the contralateral insular cortex, but not in the striatal and extended amygdala areas with extensive ipsilateral inputs. Scale bars for all panels: Large image,1 mm; inset, 250 um. **(b)** Labeled nuclei are visible bilaterally in the cortical areas adjacent to the rhinal fissure, but not in the posterior BLA or the amygdalohippocampal area that show dense ipsilateral inputs. **(c)** Labeled nuclei are visible bilaterally in the parabrachial nucleus.

**
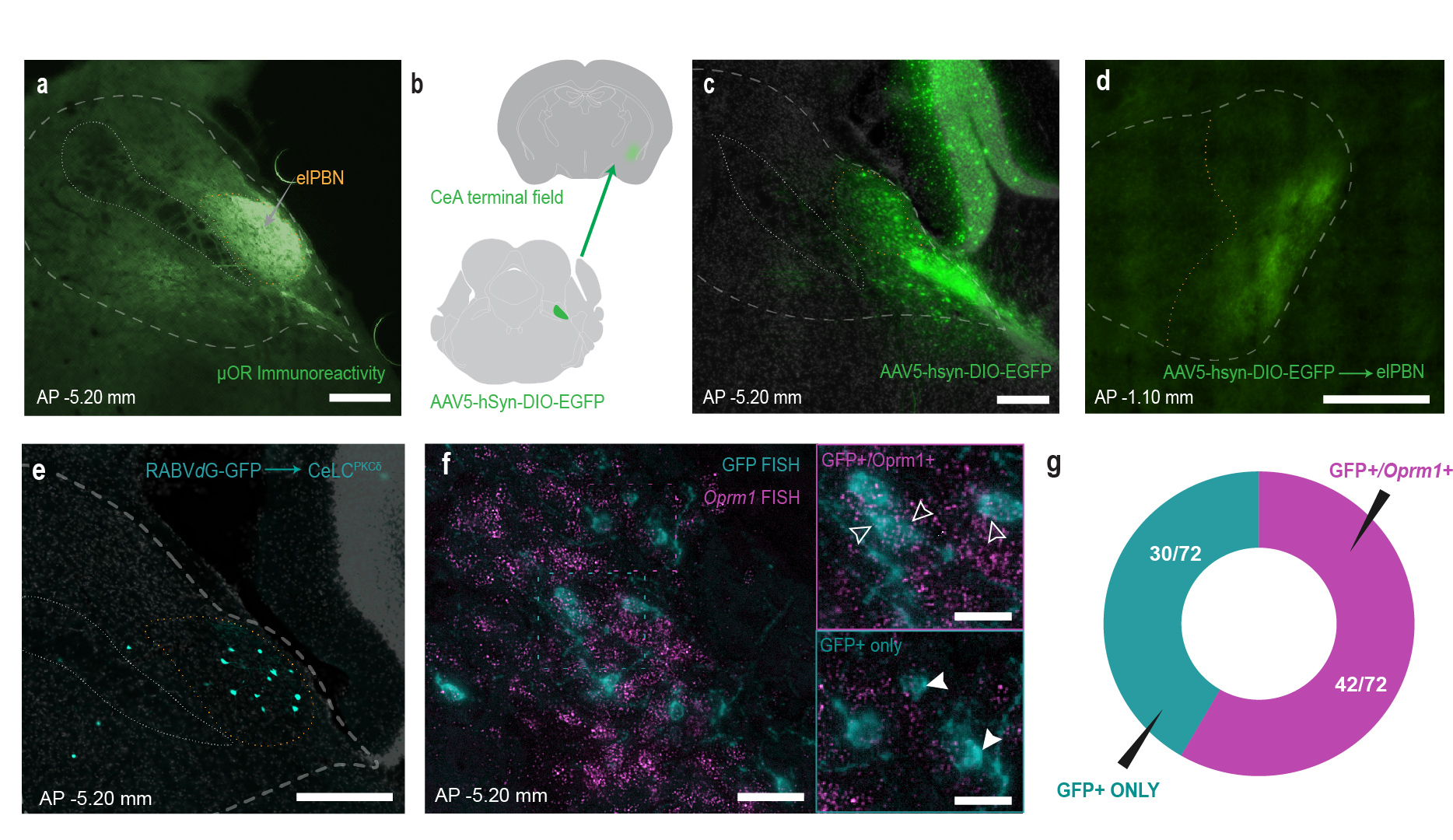
**

**Figure S10. Opioid-sensitive neurons in the parabrachial nucleus project to CeLC^PKCδ^ neurons. (a)** MOR immunoreactivity in the parabrachial nucleus, especially its external lateral nucleus (elPBN; orange). Scale bars**:** 250 um **(b)** Experimental design. We injected the elPBN of *Oprm1*-Cre mice with AAV5-hsyn-DIO-EGFP, which allowed us to visualize *Oprm1+* cell bodies in the parabrachial nucleus (PBN^MOR^) and their terminals in the CeA. **(c)** Cre-dependent EGFP expression in the parabrachial nucleus of an *Oprm1*-Cre mouse. Scale bars: 250 um **(d)** PBN^MOR^ terminals are restricted to the capsular CeC in the anterior part of the CeA. **(e)** Expression of RABVdG-GFP (Cyan) in the elPBN. We injected Cre-dependent helper AAVs, followed by RABV*d*G-GFP, into the CeA of *Prkcd*-Cre mice. Scale bars: 250 um. **(f)** *Oprm1* mRNA (magenta) colocalizes with RABV*d*G-GFP (cyan) expression in the elPBN of *Prkcd*-Cre mice, suggesting that PBN^MOR^ neurons send monosynaptic inputs to CeLCPKd neurons. Scale bar: 250 um; insets: 100 um. Filled arrowheads: GFP+/*Oprm1*- neurons; open arrowheads: GFP+/*Oprm1*+ neurons **(g)** quantification of **(f).** n=5 mice, 5 slices/mouse, 72 identified GFP+ cells after RNAScope.

***Supplementary Materials and Methods***

**Animals.**

All experiments described here were approved by the University of Pennsylvania Institutional Animal Care and Use Committee and performed in accordance with the National Institute of Health (NIH) guidelines for animal research. Male and female mice between 3-5 months of age were housed in a temperature- and humidity-controlled vivarium on a 12hr:12hr reverse light/dark cycle (lights off at 9:30AM). All experimental procedures took place during mice’s dark phase under red light. Mice were housed in groups of 2-5 prior the beginning of fentanyl treatment, at which point they were individually housed (for experiments in **Fig. 1** and **Fig. 4** where dosage is reported) or housed in groups of 2-3 (all other experiments). Housing conditions were identical for all mice in a given experiment, to avoid introducing social isolation as an additional variable. Except where noted, all experiments were performed in transgenic *Prkcd*-Cre mice (Tg(Prkcd-glc-1/CFP,-cre)EH124Gsat, MGI ID: 3844446), which were obtained from Drs. Matthew Hayes and Tito Borner. For experiments in Figs. 6D-F, *Oprm1*-Cre mice were donated by Dr. Richard Palmiter (B6.Cg-Oprm1tm1.1(cre/GFP)Rpa/J, Jackson Laboratories, strain #035574). All mice in the present studies were bred in-house from a male mouse heterozygous for the Cre transgene crossed to a female C57bL/6J mouse (Jackson Laboratories, strain # 000664). Experiments included heterozygous male and female offspring from these crosses. Food and water were available *ad libitum* throughout all experiments; during fentanyl treatment, food and fentanyl-treated water was available *ad libitum*. All data collection involving mouse handling was performed by female experimenters (LMW, AYJ, JWKW, or AML) [1].

**Experimental model of fentanyl dependence.**

The standard water bottle for each cage was replaced with a 15 mL Amuza Drink-O-Measurer bottle with a double ball-bearing sipper tube, marked by the manufacturer in 1 mL increments (Amuza Drink-O-Measurer). On day 0, the water of fentanyl-drinking mice was replaced with animal facility tap water treated with 0.02 mg/mL fentanyl citrate (Covetrus, item # 055012), modified after previous studies using fentanyl-treated water as a means of unsignaled drug delivery (**Fig. 1a**) [2,3]. Water-drinking control mice drank untreated facility tap water from identical bottles. For experiments where intake is reported, mice were weighed daily at 1:00 PM, and the intake volume was measured to the nearest 0.5 mL to calculate daily consumed dose. Bottle solutions were replenished daily. For experiments where intake is not reported, bottle volume was checked daily and replenished as needed. After 8 days, all bottles were replaced with standard animal facility water bottles containing untreated water either immediately following opioid receptor antagonist administration (**Figs. 1a and 3a**) or 20-24 hours prior to spontaneous withdrawal assessments (**Fig. 4a, 4i**).

**Drug Preparation.**

For oral fentanyl administration, fentanyl citrate (50 ug/mL, Covetrus, item #055012) was diluted in animal facility tap water to a final concentration of 0.02 mg/mL and stored at room temperature for no more than 4 weeks. Fentanyl citrate was diluted in 0.9% sterile saline and delivered at a dose of 0.2 mg/kg for acute treatment in **Fig. 3**. Naltrexone hydrochloride (Hellobio, cat #HB2452) and naloxone hydrochloride (Hellobio, cat #HB2451) were dissolved in saline and stored at 4°C for up to a year. Meloxicam (Loxicom, Midwest Veterinary Supply, cat # 515.50000.3) was diluted in saline and stored at room temperature for up to a month. Pentobarbital (Fatal Plus containing 390 mg/mL pentobarbital, Covetrus, cat # 035946) was stored at room temperature for up to a year and delivered undiluted at a volume of 0.08 mL. Unless otherwise noted, all drugs were delivered via subcutaneous injection at a volume of 10 mL/kg.

**Viral vectors.**

Viral vectors were diluted to their final working concentration in sterile phosphate-buffered saline (PBS), where necessary. Vectors were stored in 10 uL aliquots of their working titer at -80°C prior to use; during use, aliquots were stored at 4°C for up to 5 days. All viruses were injected at a flow rate of 100-125nL/min to the right central amygdala (stereotaxic coordinates: anterior-posterior AP -1.05 or -1.10 mm, medial-lateral ML +2.78 mm, dorsal-ventral DV -4.82 mm relative to bregma) or the right parabrachial nucleus (stereotaxic coordinates: AP -5.33mm, ML -1.10mm, DV -3.65mm relative to bregma) due to previous reports of lateralized function of the central amygdala in response to aversive stimuli [4–6]. For photometry experiments, 500 nL of AAV9-hsyn-FLEX-GCaMP6f (titer: 2.8x10^12^ vg/mL; Addgene, Plasmid #100837) was injected into the right CeA of *Prkcd*-Cre mice. For chronic inhibition experiments, 300 nL of AAVDJ-CMV-Kir2.1-zsGreen (titer: 1.35x10^12^ vg/mL; from Dr. Marc Fuccillo) or AAV5-hsyn-DIO-EGFP (titer:1.3x10^12^ vg/mL; Addgene, Plasmid #50457) was injected in *Prkcd*-Cre mice. For monosynaptic input tracing experiments, AAV2-hsyn-TCB-mCherry (1.7x10^12^ cfu/mL; from Dr. Kevin Beier) and AAV8-hsyn-G (1.0x10^12^ cfu/mL; from Dr. Kevin Beier) were mixed 1:1 and injected into the right CeA of *Prkcd*-Cre mice at a volume of 500 nL. RABV*d*G-GFP (8x10^8^ cfu/mL; from Dr. Kevin Beier) was injected 3 weeks later at the same location, at a volume of 500 nL. For labeling of *Oprm1*+ and *Oprm1*- inputs to the CeA, AAVrg-EF1a-Nuc-fl(mCherry)-EGFP was injected at a volume of 250 nL in *Oprm1*-Cre mice (2.5x10^13^ vg/mL; Addgene, Plasmid #112677).

**Behavioral testing.**

*Prkcd*-Cre mice were used in all behavior experiments, and both males and females were included in all experimental groups. Mice were habituated to behavioral apparatuses for 1 hr each for the 2 days prior to the beginning of behavioral testing and habituated to the apparatus for 30 minutes with the experimenter present before beginning testing each day. Mice were returned to the colony room after all animals in the group had completed behavioral testing each day. Males and females were not tested in the same room at the same time. All experiments took place between 10:30AM-6:30PM.

*Apparatus.*

For all experiments, mice were placed in individual enclosures on a custom-made elevated rack with a perforated metal floor (61 cm x 26 cm; **Fig. S5a**). For fiber photometry experiments (**Fig. 2, 3)** the enclosures were 7 cm diameter x 10 cm height red Plexiglas cylinders with a custom-printed, form-fitting lid containing a passage for the photometry patch cable. For experiments conducted in **Fig. 4**, the enclosures were 16 cm x 16 cm x 38 cm height plexiglas rectangular enclosures with a black foam lid. These apparatuses accommodated both withdrawal scoring and nociceptive testing without requiring movement of the animal. Unless otherwise indicated, all stimuli were applied to the left hindpaw, contralateral to the right CeLC viral injection.

*Withdrawal assessment.*

Naltrexone-precipitated withdrawal was assessed from 15-30min following naltrexone administration, in the afternoon of Treatment Day 8. (**Fig. 1a, b**) Naloxone-precipitated withdrawal was assessed from 0-15min following naloxone administration on the afternoon of Treatment Day 8 (**Fig. 3a, b**). These timepoints were chosen to match the pharmacokinetic profiles of the antagonists and the cell activation-related endpoints of interest. Spontaneous withdrawal was assessed 20-24 hrs following the removal of fentanyl-treated water **(Fig. 4a, 4b, 4i, 4m).** A Logitech webcam (C920x HD Pro) protected by a custom Plexiglas enclosure (58 cm x 32 cm x 20 cm) were used to record somatic signs from the underside of the mouse (**Fig. S5a**); videos were scored offline using BORIS behavioral software[7] by an experimenter blinded to experimental condition. To score each video, mice were continuously observed for 15min and the total instances of genital grooming, digging at the corners of the apparatus, bouts of running, jumping, paw tremors, head shakes, wet dog shakes, and the total number of observed fecal boli were recorded. In addition, the presence/absence of a resting tremor and teeth chattering were recorded once for each minute they were observed. All instances and presence of events (1 point per minute present) were summed to get the global withdrawal score for each animal. Because other research groups have observed affective and somatic opioid withdrawal signs existing independently,[8,9] behaviors were further divided into three subscores: an autonomic score (sum of fecal boli and minutes with teeth chattering), affective score (genital grooming, running, jumps, digging) and shakes/tremors score (paw tremors, head shakes, wet dog shakes, minutes with resting tremor). To our knowledge, this is the first report that divides withdrawal signs in this way. Ethovision XT (Noldus, version 15) was run on the same 15 min video to calculate the distance traveled for each mouse.

*Von Frey Up-down*

TouchTest sensory evaluator filaments (North Coast Medical and Rehabilitation Products) of logarithmically increasing thickness (range: 0.07-6 g, initial filament: 0.16 g) were sequentially applied to the left hindpaw until they bent slightly. The force necessary to produce a hindpaw withdrawal response was calculated using the Up-Down method as described previously[10,11] and reported as 50% withdrawal threshold.

*Warm-water tail withdrawal*

A Sous-vide Precision Cooker Nano 2.0 (Anova) maintained a water bath at 45°C, 48°C, or 50°C; we chose these temperatures to engage different combinations of transient receptor potential channels that control innocuous and noxious heat sensation [12]. Mice were removed from the apparatus and briefly restrained in a cotton towel. The distal 1-2 cm of their tail was submerged in the water bath. The tail was immediately removed once it curled or flicked, and the latency to do so was recorded. If the mouse did not withdraw its tail within 20 sec, it was removed to avoid tissue injury, and the latency was recorded as 20 sec. The mouse’s tail was dried with a paper towel, and the mouse was returned to the apparatus. Temperatures were tested from lowest to highest, and a minimum of 15 min elapsed between consecutive tests.

*55°C hot water drop.*

A Fisher Micro hotplate (Thermo Fisher Scientific) was used to maintain approximately 50 mL of water at 55°C. Water droplets were expressed from the end of a 1 mL syringe and applied to the plantar surface of the left hindpaw while the mouse was on the apparatus. The mouse’s response was video-recorded and scored offline using BORIS behavioral software[7] by an experimenter blinded to experimental condition. Total time spent in affective behaviors (attending the paw, licking the paw, guarding the paw, rearing, running, and jumping) were scored for the 30 sec immediately following the hot water application.

*50°C inescapable hotplate.*

Mice were removed from the apparatus and placed on an enclosed hotplate (Bioseb hot and cold plate; 16.5 cm x 16.5 cm floor) maintained at 50°C. The mice were promptly removed after 60 sec to avoid tissue damage and immediately returned to their home cage. Cameras (Logitech C920x HD Pro Webcam) were set up on either side of the hotplate to videorecord behavior while the mouse moved freely on the plate. Videos were scored offline using BORIS behavioral software [7] by an experimenter blinded to experimental condition. Instances of paw flicks, hops, and jumps, and the duration of attending behaviors (licking, attending, or guarding of one or more paws), and escape behaviors (rapid rearing, running, hopping, and jumping), were scored for the full 60 sec hotplate exposure.

**Two-bottle choice.**

Mice were individually housed in standard cages with a 3D-printed two-bottle choice apparatus equipped with two 15 mL conical tubes modified as sipper bottles [13]. One tube contained untreated tap water from the animal facility, and the other contained the same water treated with 0.02 mg/mL fentanyl citrate. The weights of the mice and the intake volume from each bottle were recorded daily at 12:00 pm for 16 days. The positions of the bottles were switched every day to minimize side preference effects.

**Immunohistochemistry.**

Formalin-fixed tissue was coronally sectioned at 30-50 um, protected from light, and stored at 4°C in PBS until free-floating immunohistochemistry was performed. Tissue was then washed in 0.5% Triton-X 100 detergent in PBS (Sigma-Aldrich) 4 times for 10 min each to permeabilize the cell membranes, rinsed 4 times for 10 min in fresh PBS, and incubated in normal donkey serum blocking solution (Jackson ImmunoResearch, 10% in 0.1% Triton-X+PBS) to block off-target antibody binding. Slices were then incubated in the primary antibody diluted in blocking solution to the desired concentration for 48 hrs at 4°C. Primary antibodies include: rabbit anti-FOS (1:1000, Synaptic systems, cat # 226008), guinea pig anti-FOS (1:1000, Synaptic systems, cat # 226308), mouse anti-PKCδ (1:1000, BD Transduction Laboratories, cat # 610397), rabbit anti-MOR (1:1000, Abcam, cat # ab134054); chicken anti-GFP (1:1000-1:2000, Abcam, cat # ab13970), rabbit anti-dsRed (1:1000, Takara, cat # 632496). Slices were then washed in PBS 4 times for 10 min, followed by incubation with secondary antibody diluted in blocking solution to the desired concentration for 24 hrs at 4°C. Secondary antibodies include: Alexafluor Donkey anti-mouse 647 (1:500, Invitrogen Thermo Fisher, cat # A31571), Alexafluor Donkey anti-guinea pig 647 (1:500, Jackson ImmunoResearch, cat # 705-605-148), Alexafluor Donkey anti-rabbit 488 (1:500, Invitrogen Thermo Fisher, cat # A21202), Alexafluor Donkey anti-mouse 647 (1:500, Invitrogen Thermo Fisher, cat # A31571), Alexafluor Donkey anti-chicken 488 (1:500, Jackson Immunoresearch, cat # 703-545-155), Alexafluor Donkey anti-chicken 594 (1:500, Jackson ImmunoResearch, cat # 703-585-155). Tissue was then washed in PBS 4 times for 10 min, then mounted onto glass slides and incubated with a 1:10,000 solution of DAPI counterstain diluted in water for 10 min (Fisher Scientific). The tissue was then covered with Fluoromount-G mounting medium (Fisher Scientific), coverslipped, and dried flat overnight in a dark, cool place before imaging.

**Fluorescent *in situ* hybridization.**

The RNAScope Multiplex Fluorescent v2 Assay (Advanced Cell Diagnostics) was used to label mRNA according to the manufacturer’s instructions. Briefly, mice were perfused as described previously, and brains were removed and postfixed in 10% normal buffered formalin for 24 hrs. The whole brains were then submerged in 10%, 20%, then 30% sucrose at 4°C until the tissue sank to the bottom of the container (approximately 24-48 hrs for each concentration). Brains were then frozen in TissueTek O.C.T. compound (Thermo Fisher Scientific) at -20°C. 16 um sections of the CeA or PBN were collected and placed onto dry SUPERFROST PLUS (Thermo Fisher Scientific) slides while still frozen. Slides were either processed immediately or stored at -80°C for up to 3 months. Slides were next washed with PBS for 1 min, then baked for 30 min at 60°C in a HybEZ oven (Advanced Cell Diagnostics). Slides were postfixed in chilled 10% neutral buffered formalin for 15min at 4°C. The slides were then dehydrated by immersion in 50%, then 70%, then 100% EtOH x 2 for 5 min at room temperature. The slides were incubated in 6% hydrogen peroxide for 10 min at RT, then washed several times in distilled water, then pretreated with RNAScope Target Retrieval Reagent heated to 99°C for 15 min, inside a commercial steamer (Oster). Slides were rinsed for 15 sec, transferred to 100% ethanol for 3 min, and dried. A hydrophobic barrier was drawn around the tissue using an Immedge pen, and the slides were dried overnight in a closed drawer at room temperature. The following day, slides were incubated in the provided Protease III reagent for 30 min at 40°C and washed twice with distilled water. Slices were next incubated in the desired mixture of cDNA hybridization probes for 2 hrs at 40°C, then washed twice in the provided Wash Buffer. Probes used included: Mm-Oprm1-C1 (Advanced Cell Diagnostics, Cat # 315841), Mm-EGFP-C2 (Advanced Cell Diagnostics, Cat #400281-c2), 4-Plex Negative control (Advanced Cell Diagnostics, Cat#321831). Amplification was performed by incubating tissue at 40°C in the provided FL v2 Amp1 (30 min), FL v2 Amp2 (30 min), then the FL v2 AMP3 (15 min), with 2 min washes in the provided wash buffer before and after each step. Slices were then fluorescently stained by first incubating sections in the provided TSA Plus HRP solution for the desired channel for 15 min at 40°C, then in a 1:5000 solution of Opal 520 or 690 fluorescent dye (Akoya Biosciences, cat # FP1487001KT; cat # FP1497001KT) diluted in the provided TSA buffer for 30 min at 40°C, then a final incubation with the provided HRP blocker for 15 min at 40°C, with 2 min washes in the provided Wash Buffer between each incubation. This process was repeated for each channel. Lastly, sections were counterstained using the provided DAPI reagent, mounted with Fluoromount-G mounting medium, and coverslipped. Pairs of sections were collected such that for each stained section, an additional section was hybridized with the 4-Plex Negative control probe, which contained bacterial RNA instead of a cDNA.

**Imaging and quantification.**

All imaging was performed using a Keyence BZ-X800 all-in-one fluorescent microscope with PlanApo-λ x4, PlanApo-λ x20, and PlanApo-λ x40 objectives. Image processing was completed for all images using the Keyence BZ-X analyzer software (version 1.4.0.1). For placement checks/viral transduction and targeting verification, slices were imaged in a single plane of focus at 4x magnification. For histological quantification, slices were imaged with 20x-magnified z-stacks or, for fluorescence *in situ* hybridization, in a single plane of focus at 40x magnification. The exposures for FITC, TRITC, and Cy5 were adjusted to avoid overexposed pixels. Exposures were standardized such that a single exposure for each channel was used across all tissue in the same experiment. For quantification of cells with a single marker protein or non-overlapping markers, quantification was performed manually using the Adobe Photoshop Counter function or semi-manually using Cellpose 2, as indicated[14]. When quantifying colocalization of 2 or more markers, as in **Fig. 1i-j,** quantification was performed first in Cellpose 2, then masks were exported as .pngs, and overlaid masks were counted using the Threshold tool in FIJI [15]. When using Cellpose 2 or 3, a single set of settings was developed and used to quantify all tissue in the same experiment.

**Stereotaxic surgery.**

Adult mice (~12 weeks of age) were anesthetized with isoflurane gas in oxygen (induction: 5%; maintenance: 1.0-2.5%) and mounted onto a stereotaxic frame (KOPF instruments). Mice were administered meloxicam (5 mg/kg, s.c.) and 1 mL saline (s.c.) at the beginning of the surgery. Eye lubricating gel (Optixcare) was applied at the beginning of surgery and every 45 min throughout, and reflexes and respiratory rates were assessed every 10 minutes to ensure a surgical plane of anesthesia. The skull was manually leveled and a ~1 mm craniotomy was drilled over the target area. A 10 uL Hamilton Nanofil syringe fitted with a 33 gauge beveled needle was slowly lowered into the right CeLC (coordinates from Bregma: AP -1.10mm, ML +2.78mm, DV -4.82mm) or the right parabrachial nucleus (coordinates from Bregma: AP -5.33mm, ML +1.10mm, DV-3.65mm), and 250-500 nL of AAV or RABV*d*G vectors containing transgenes of interest were infused at a rate of 100-125nL/min. All injections were performed unilaterally in the right hemisphere because of previous reports of lateralization in central amygdala function in response to aversive stimuli and in the maintenance of aversive states [4,5,16]. Incisions were closed with 5-0 Vicryl sutures (Ethicon).

For fiber photometry experiments, immediately after virus injections the skull was scored with a scalpel blade and 2 small screws (~1.7 mm diameter, 1.5 mm length) were placed distal to the craniotomy as additional anchor points. Immediately after the viral injection, an optical fiber (5.0mm fiber, 400 um diameter, Doric Lenses) was slowly lowered to approximately 0.2mm over the target coordinate (in the DV plane) and fixed to the skull using MetaBond (Parkell) followed by Jet Set dental acrylic (Lang Dental) to create a reinforced headcap and cover exposed skull. The dental acrylic was mixed with black iron oxide power to reduce the chance of any excitation or emission light leak.

Following all surgeries, mice recovered under a heat lamp until they fully regained the righting reflex, and were then returned to their homecage. Mice were monitored daily for 3 days following surgery to ensure integrity of the sutures and headcaps, and meloxicam was administered again 24 hrs after surgery for viral injections and 24, 48, and 72 hrs after fiber implant to minimize pain and brain inflammation.

***withdrawal*FOS induction and immunohistochemistry.**

Male and female *Prkcd*-Cre mice were individually housed on day 0 of fentanyl or water administration. On Treatment Days 6 and 7, mice were habituated in their home cages to the testing room for 2 hrs daily. On Day 8, mice were habituated in their home cages to the testing room for a minimum of 30 min. Mice were then injected with naltrexone (1 mg/kg, s.c.) or saline and immediately returned to their homecage. The fentanyl-treated water was removed and replaced with untreated water. We waited 15 min for naltrexone to take effect, then an additional 90 min for the peak of FOS protein production. Thus, 105 min after naltrexone or saline injection, mice received an overdose of pentobarbital (Fatal Plus, 0.08 mL, i.p.) and were placed back in their homecage until toe-pinch and corneal reflexes were absent. Mice were then promptly transcardially perfused with phosphate-buffered saline followed by 10% normal-buffered formalin. The brain was removed and postfixed in 10% normal-buffered formalin overnight, then incubated in 30% sucrose in PBS until the tissue sunk. 30 um coronal slices through the expanse of the central amygdala were collected on a cryostat (CM3050S, Leica Biosystems). Every 3^rd^ 30 um slice was stained for FOS and/or PKCδ using immunohistochemistry (see section for details). The anterior-posterior coordinate was determined for each slice and borders were drawn based on the Paxinos and Watson 5^th^ edition stereotaxic atlas [17], and the CeA was imaged at 20x magnification with z-stack. FOS+ and/or PKCδ+ neurons were quantified in each of the CeA subnuclei from each side of one slice per 0.2 mm. Quantification was performed using CellPose 2.0 and ImageJ (FIJI; **Fig. 1, Fig. S2)** [18,19]. Except where noted, each point represents the sum of the cells in the left and right hemisphere.

**In vivo Fiber photometry.**

500 nL of AAV9-hsyn-FLEX-GCaMP6f-WPRE-SV40 was injected into the right CeA of male and female *Prkcd-*Cre mice (AP -1.10, ML +2.78, DV -4.82) and a 400 um borosilicate fiber-optic cannula was implanted 200 um above the injection site (5 mm length, 0.66 NA; Doric Lenses; see surgery section for details). A minimum of 3 wks of surgical recovery elapsed between surgery and the beginning of habituation. For two days prior to the first test day, and on each test day, mice were habituated to the testing room in their home cage for 1 hr without the experimenter present, then to the apparatus and patch cord for 15 min with the experimenter present.

Optical recordings of GCaMP6f fluorescence were acquired using an RZ10x fiber photometry detection system (Tucker-Davis Technologies), a processor with Synapse software (Tucker-Davis Technologies), and optical components (Doric Lenses and ThorLabs). LED-generated light was filtered through a fluorescence minicube at spectral bandwidths of 460 nm and 405 nm and passed through a pre-bleached, low auto-fluorescence mono fiber-optic patch cord (Doric Lenses) connected to the external portion of each mouse’s fiber-optic cannula via a zirconia mating sleeve (Doric Lenses). The power output at the tip of the patch cable was adjusted daily to ~50uW for the 460 nm channel (calcium-dependent signal; modulated at 210 Hz), and to ~15uW for the 405 nm channel (isosbestic control; modulated at 330 Hz). Signals were low-pass filtered at 6 Hz. All recordings utilized both channels.

On the first (baseline) test day, 4 stimuli were presented to all mice for 10 trials each with a 90 sec intertrial interval (ITI): 0.16 g von Frey Filament (TouchTest), 25-gauge needle “pinprick”, 55°C hot water drop, all delivered to the left hindpaw; and an airpuff from a condensed air canister delivered 1 cm from the left cheek/eye (**Fig. 2a**). A TTL pulse was delivered to the Synapse software by the experimenter at the moment of stimulus application by means of a custom-built trigger button. Trials were aborted if the mouse responded behaviorally to the approach of the stimulus. Additionally, trials were excluded if the stimulus did not contact the mouse or produce the expected behavioral responses. Between each stimulus type, the recording was turned off for 5 min to minimize photobleaching.

On both the acute fentanyl and withdrawal test days, fluorescence was recorded for 7 min to establish a baseline period prior to injection (**Fig. 3a**). While continuously recording, the mouse was removed and injected with either fentanyl (0.2 mg/kg, s.c.) or 0.9% saline for the acute fentanyl test in naïve mice (**Fig. 3a**), or naloxone for the withdrawal test (3 mg/kg, s.c), and returned to the enclosure, all while still attached to the patch cord. A TTL pulse was delivered immediately before the removal of the mouse, and immediately after the return of the mouse to the enclosure (the “injection period”). Fluorescence was then recorded for 15 min. Following a 5 min rest in the recording, mice were again presented with the highly-salient stimuli, the 55°C hot water droplet and the airpuff. To ensure all testing occurred while fentanyl or naloxone was at its peak effectiveness, only 5 trials of each stimulus were delivered, again with a 90 sec ITI. At the end of the last recording, mice were immediately disconnected from the patch cable and returned to their homecage. Behavioral responses to each stimulus were scored for the first 30 sec post-delivery using BORIS. All behaviors were recorded, including flinching the paw, startle, jumps, attending to the stimulus (orienting to the paw or to the grate where the stimulus came from), guarding the paw (lifting, hiding, or otherwise protecting the paw), licking the paw, rearing, and running.

At the completion of experiments, all mice were perfused and immunohistochemistry was performed to amplify GCaMP6f fluorescence using chicken anti-GFP and donkey anti-chicken AF488 antibodies. Each brain was imaged, and animals without virus expression and proper fiber placement in the CeA were excluded from the experiment. In addition, two animals were excluded only from the withdrawal recording: one female mouse in the fentanyl-drinking group was excluded and euthanized because of a sudden health decline; and one male mouse in the water-drinking group was excluded from the withdrawal recording because after data processing it was apparent that the patch cord had lost contact the fiber during the naloxone injection.

**Fiber photometry data.**

Data from the Synapse software was processed using an independent deployment of the open-source pMAT fiber photometry analysis software and MATLAB[20]. For stimulus-locked recordings, peri-event time histograms were generated in pMAT from -10 sec to 10 sec relative to the TTL pulse for each trial, with a baseline sampling window from -10 sec to -5 sec, and both the trial-based dF/F and z-scores were exported. All trials of a given stimulus were averaged for a given mouse; therefore, each data point is one mouse. The max amplitude of the Ca^2+^ responses in the first 5 sec following the stimulus (peak z-score) were determined from the mean trace, and the net AUC of the mean dF/F trace or z-scored dF/F was calculated in Graphpad Prism for the 5 sec following stimulus presentation. These AUCs were statistically compared to baseline areas under the curve calculated from -10 sec to -5 sec relative to the stimulus, to minimize the influence of Ca^2+^ responses related to the experimenter approaching the mouse. For the acute fentanyl and withdrawal tests, we further adjusted the ~25 min drug injection recording to account for the persistent downward trend of the signal due to photobleaching. The entire recording and the timestamps for the TTLs around the injection were exported into MATLAB, the signals of each channel was smoothed and downsampled from 10 Hz to 3 Hz, and the 465 nm signal recording for the pre-drug baseline was re-fit to the same period of the smoothed 405 nm isosbestic recording using custom MATLAB code. The dF/F was re-calculated from this smoothed and downsampled signal, and a baseline z-score for each timepoint was calculated from this data using the 7 min pre-injection period as the baseline window.

**Viral-mediated overexpression of Kir2.1.**

300 nL of AAVDJ-CMV-DIO-Kir2.1-zsgreen or AAV5-hsyn-DIO-EGFP was injected into the right CeA of male and female *Prkcd*-Cre mice. 2.5-3 wks later, mice underwent baseline testing to determine values for mechanical response thresholds (von Frey up-down), thermal reflexive thresholds (warm water tail withdrawal at 45°C, 48°C and 50°C), and responses to a 55°C hot water drop applied to the hindpaw, all as described above (**Fig. 4i**). After a 30min period to habituate to the testing apparatus, mice underwent the von Frey up-down procedure on the left, then right paw. Next, a hot water drop was applied to the left, then right paw. Tail flick latencies were then collected in order of increasing temperature. A minimum of 15 min elapsed between each trial. Mice were returned to their homecage immediately after the 50°C tail withdrawal test. After baseline testing, mice began the 8-day fentanyl or water drinking procedure. All Drink-o-measurer bottles were removed late afternoon on the 8^th^ drinking day and replaced with regular animal facility water bottles. On Day 9, 20-24 hrs after bottle removal, mice were returned to the same testing room and were tested on all nociceptive procedures in the same sequence as on the baseline day. Video recordings of the mice were also collected during the habituation period, and behavior from the first 15 min of the period were scored offline to obtain the global withdrawal score and distance traveled. Following the conclusion of the experiments, mice were perfused, and 50 um slices were collected through the anterior-posterior axis of the CeA of each animal to confirm viral transduction and targeting. Data from mice in which few cells were visible in the CeA or with significant additional off-target expression were excluded.

**Monosynaptic rabies tracing.**

500 nL of a 1:1 mixture of AAV2-DIO-TCB-mCherry and AAV8-FLEX-RABV*d*G was injected into the right CeA of male and female *Prkcd*-Cre mice (**Fig. 5a**). Two weeks later, the same area was injected with 500 nL of RABV*d*G-GFP. Five days later, mice were perfused and the brains were postfixed and cryoprotected. Consecutive 50 um slices were collected from the entire brain. Every 2^nd^ 50 um slice from the central amygdala was imaged at 20x to confirm the presence of putative starter cells. We then selected areas of interest from an initial scan of the tissue. We imaged the right hemisphere of every 2^nd^ slice from +0.70 mm AP to -4.20 mm AP, and from -4.70 mm to -5.80 mm AP at 20x magnification with z-stacks. GFP+ input cells were counted manually in FIJI [15] using the Cell Counter tool, and the number of cells expressing both mCherry and GFP (i.e. putative starter cells) were counted in every 2^nd^ CeA slice using HALO-AI. The x-y coordinates of each identified GFP+ cell were exported from FIJI, and cells/slices were aligned to the Kim Lab Unified Anatomical Atlas [21] using the open-source, MATLAB-based atlas registration program SHARCQ [22]. Cells that were identified in individual subnuclei of brain structures were summed to result in a single number for each macrostructure according to the headings present in the Unified Anatomical Atlas.

In a separate group of RABV*d*G-injected mice, 50 um slices were collected from the central amygdala to verify starter cell expression, then 5 pairs of 16 um slices were collected from the parabrachial nucleus of each mouse. RNAScope was performed as described, to stain for *Oprm1* mRNA (using Mm-Oprm1-C1 cDNA probe and Opal 690 fluorescent dye) and to simultaneously amplify the EGFP in the RABV*d*G vector (using Mm-EGFP-C2 cDNA probe and Opal 520 fluorescent dye). The number of GFP+ input cells with and without *Oprm1* mRNA colocalization in the right PBN were manually quantified using the Counter tool in Photoshop (Adobe).

**Identification of opioidergic inputs to the central amgydala.**

250 nL of AAVrg-EF1a-Nuc-fl(mCherry)-EGFP was injected into the right CeA of male and female *Oprm1-*Cre mice (**Fig. 5d**). Eight weeks later, mice were administered pentobarbital (Fatal Plus, 0.08 mL, i.p.) and monitored until the toe pinch and corneal reflex were absent. Mice were then transcardially perfused, brains were dissected and post-fixed, and 50 um slices were collected every 2 mm throughout the brain. Both the mCherry and GFP fluorescent signals were amplified using immunohistochemistry (antibodies: rabbit anti-dsRed and donkey anti-rabbit 594; chicken anti-GFP and donkey anti-chicken 488). Regions we had previously identified as having dense inputs to CeLC^PKCδ^ neurons with the RABV*d*G study were imaged as 20x-magnified z-stacks. Regions were then manually outlined in each slice on which they appeared using Photoshop, and the number of GFP+ and mCherry+ cells were counted using Cellpose 2.0. [18,19] All GFP+ and mCherry+ cells from each slice in which they appeared and from each animal were summed to determine total % *Oprm1*^+^ inputs (i.e., 100 * (GFP^+^ cells/mCherry^+^ + GFP^+^ cells)).

In a separate group of *Oprm1*-Cre mice, 500 nL of AAV5-hsyn-DIO-EGFP was injected into the right PBN (-5.4mm AP, +1.35mm ML, -3.65mm DV). Mice in this study also received a unilateral injection of AAV9-hsyn-DIO-mCherry in the right CeA (not shown). 6 weeks later, mice were administered pentobarbital (Fatal Plus, 0.08 mL, i.p.) and monitored until the toe pinch and corneal reflex were absent. Mice were then transcardially perfused, brain tissue was collected and post-fixed, and every 2^nd^ 50 um slice was collected through the CeA and the PBN. Representative images were taken of putative PBN^MOR^  cell bodies in the PBN (i.e., EGFP+ neurons) and EGFP+ terminals in the CeA as 20x-magnified z-stacks.

**Statistical analyses.**

Power analyses were conducted in G*Power[23] at the beginning of each experiment to determine minimum sample sizes and adjusted as needed based on observed effect sizes. Male and female mice were used in every experiment; unless otherwise noted, analyses were conducted with sex as an additional factor, and results with positive main or interaction effects of sex are reported. Unless otherwise noted, data are presented as the mean +/- standard error of the mean, with gray circles representing the observed value for an individual subject. Kolgomorov-Smirnoff tests were used to assess the normality of data sets, and two-tailed paired or unpaired t-tests, Mann-Whitney tests, One- and Two-Way ANOVAS, or mixed model analyses were calculated as indicated using Prism software (Graphpad, v9). Paired t-tests or repeated-measures ANOVAs were used when comparing subjects’ results to their own baselines. Where ANOVA or mixed model analyses showed significant main effects or interactions, *p*-values for the relevant comparisons were adjusted using Bonferroni’s method.

***Supplementary Results***

**Characterization of fentanyl drinking under 24hr homecage forced access and two-bottle choice conditions.**

To render male and female mice fentanyl-dependent, the homecage water supply was replaced with water treated with 0.02 mg/kg fentanyl for 8 days (**Fig. 1a**). Control mice drank untreated water. We used *Prkcd-*Cre mice, which stably express Cre recombinase under the control of the promoter for PKCδ, to gain genetic access to CeLC^PKCδ^ cells in later experiments, and so we also assessed our model in *Prkcd­*-Cre mice. Fentanyl-drinking mice consumed a similar volume of water over the 8-day period to water-drinking mice (**Fig. S1a**). There was a significant effect of Treatment Day and subject on intake volume, with both water- and fentanyl-drinking mice consuming a significantly higher volume on Treatment Day 7, and fentanyl-drinking mice consuming significantly more on Treatment Days 6 and 8, compared to day 1. Neither male nor female mice in either experimental group experienced significant weight loss during the 8-day administration paradigm, indicating that they received sufficient hydration (**Fig. S1b**). While doing so, fentanyl-drinking mice consumed an average of 3.99 mg/kg/day fentanyl (**Fig. S1c**). In line with the increased intake of fluid over the course of the experiment, mice consumed more fentanyl on Treatment days 4, 7, and 8 compared to Treatment Day 1. Over the 8-day experiment, female mice consumed more than male mice **(Fig. S1d),** with female but not male mice showing an increased intake of fentanyl from Treatment day 1 to Treatment day 8 (**Fig. S1e**). However, we noticed similar patterns of drinking in water-drinking mice, suggesting that this result was due to the experimental conditions rather than representing volitional dose escalation. In support of this conclusion, this drinking procedure did not produce a significant preference for fentanyl-treated vs. untreated water in a two-bottle choice procedure: on average, mice consumed similar quantities from both bottles throughout treatment (**Fig. S1f-h**). Importantly, the mean intake across the experiment for individual animals directly correlated with their global withdrawal scores (**Fig. S1i**), as did mice’s individual intake on Treatment day 8. (**Fig. S1j**)

**Additional anatomical characterization of *withdrawal*FOS in the CeA.**

Consistent with our initial observations, we found that the magnitude of the increase in FOS expression was higher in the CeC and CeL for every fentanyl-drinking, naltrexone-treated mouse compared to the CeM (**Fig. S2a**). This effect was also significant when the CeLC as a whole was compared to the CeM. (**Fig. S2b**).

Evidence suggests that the right hemisphere CeA plays a greater role in nociceptive processes, contributing more to the generation of pain-related aversive behaviors and responses to nociceptive stimuli than the left [4,5,16,24]. We hypothesized that aversive opioid withdrawal would also result in increased withdrawal-induced FOS expression in the right CeA. However, we did not detect a significant main effect of hemisphere for the CeA as a whole (**Fig. S2c**) or for any subregion (**Fig.** **S2d-g**). There was a significant interaction of hemisphere x Drinking condition x Withdrawal condition in the CeM, but no pairwise comparisons were significant after adjusting for multiple comparisons (**Fig. S2f**). Therefore, while there were subtle differences in the left and right side of the CeA in terms of *withdrawal*FOS expression, specifically in the CeM, we did not detect a robust lateralization effect, such as those reported previously in the context of pain [4,5,16,24].

**Sex differences in CeLC^PKCδ^ neural activation during fentanyl withdrawal**

The CeA is a sexually dimorphic structure with respect to specific functions (e.g. CGRP signaling-mediated pain processing,[25,26] response to chronic alcohol[27]). For this reason, we assessed whether there were sex differences in PKCδ activation during opioid withdrawal. Though there was a significant Sex x Drinking Condition interaction on the proportion of FOS+ cells that co-express PKCδ**,** no pairwise comparisons were significant after correcting for multiple comparisons. (**Fig. S2h**). However, we did find that there were sex differences in the percent of PKCδ^+^ neurons that were activated under each condition. While there was no main or interaction effect of sex, pairwise comparisons showed that naltrexone significantly elevated the % of PKCδ^+^ neurons colocalized with FOS in water-drinking male, but not female, mice. (**Fig. S2i**) Additionally, naltrexone did not further elevate FOS colocalization in PKCδ^+^ neurons in fentanyl-drinking male mice.

**Behavioral characterization of spontaneous fentanyl withdrawal**

On Treatment Day 8, fentanyl-treated water was replaced with untreated water for fentanyl-drinking mice. We then assessed spontaneous withdrawal 20-24 hrs later, on Day 9. *Prkcd*-Cre mice exhibited typical signs of opioid withdrawal, with fentanyl-drinking mice displaying significantly higher global withdrawal scores than water-drinking mice (**Fig. 4b**). This effect was present in both sexes, and we did not detect a significant effect of sex on global withdrawal scores (**Fig. S5b**). Others have observed previously that affective and somatic opioid withdrawal signs can exist independently of one another during acute withdrawal. To determine which withdrawal signs drive the global withdrawal scores in our model, we calculated subscores based on three broad categories of typical opioid withdrawal, present in both people and rodent models: shakes and tremors, autonomic symptoms, and affective symptoms (**Fig. 4c-e**). To our knowledge, this is the first use of this division to describe opioid withdrawal scores. In our model, fentanyl withdrawal was characterized by a significant increase in the number of shakes and tremors (**Fig. 4c**), including significant increases in resting tremor, body shakes, head shakes, and paw tremors (**Fig. S5c-f**). In addition, fentanyl-drinking mice exhibited more autonomic withdrawal symptoms than water-drinking mice (**Fig. 1d**): they excreted significantly more feces (**Fig. S5g**) and exhibited more frequent teeth chattering (**Fig. S5h**). Overall, fentanyl-drinking mice did not exhibit significantly more affective behaviors than water-drinking mice (**Fig. 4e**). They did spend more time in genital grooming, a frequently-reported withdrawal-associated behavior, (**Fig. S5i**) but we did not detect significantly more jumping (**Fig. S5j**), defensive treading (**Fig. S5k**), digging (**Fig. S5l**), or limb biting (**Fig. S5m**). Fentanyl-drinking mice also displayed significantly lower locomotor activity during the 15min spontaneous withdrawal period (**Fig. 4f**). Thus, our fentanyl-drinking paradigm is sufficient to produce dependence in male and female *Prkcd*-Cre mice, indicated by the development of the spontaneous withdrawal syndrome after the removal of fentanyl.

Chronic opioid treatment can produce paradoxical hyperalgesia[28,29]. In addition, new or returning pain can accompany opioid withdrawal in clinical populations [29,30]. We therefore assessed nociceptive sensory-reflexive thresholds and affective-motivational behaviors in response to mechanical and thermal stimuli, both on Treatment Day 6 of fentanyl and immediately after the spontaneous fentanyl withdrawal assessment (**Fig. S6a**). We did not detect an effect of treatment condition or experimental timepoint on 50% withdrawal thresholds in the von Frey Up-Down assay (**Fig. S6b**). While we detected a significant effect of experimental timepoint on tail withdrawal latency from 48°C and 50°C water, wherein a gradual decline in withdrawal latencies occurred over the 3 Treatment Days, multiplicity-adjusted tests did not detect any significant differences in fentanyl-treated mice between timepoints for either water temperature (**Fig. S6c**). We also did not see an effect on the time mice engaged in affective responses to a hot water drop applied to the left hindpaw (**Fig. S6d**) or an inescapable hotplate (**Fig. S6e**). Thus, our fentanyl-drinking procedure does not produce robust fentanyl-induced hyperalgesia or withdrawal-induced hyperalgesia.

**Parabrachial MOR-expressing neurons project directly to CeLC^PKCδ^ neurons.**

CeLC^PKCδ^ neurons are the ascending target of the spinoparabrachial pathway, and the parabrachial nucleus🡪CeLC input has a well-established role in processing and responding to noxious and non-noxious aversive stimuli.[5,31–33] Additionally, the parabrachial nucleus, and particularly its external lateral subnucleus, expresses MORs at high density (**Fig. S10a**). Our viral tracing indicates that *Oprm1*+ neurons in the PBN project to the CeA, but it is unclear whether *Oprm1*+ neurons synapse on CeLC^PKCδ^ neurons. To address this, we more thoroughly characterized the inputs from the parabrachial nucleus to the CeA and to CeLC^PKCδ^ neurons in particular. Many subnuclei of the PBN express MORs, but they are particularly densely expressed in the external lateral region of the PBN (elPBN; **Fig. S10a**). As expected, [34] the injection of a virus containing a Cre-dependent fluorophore into the PBN of *Oprm1*-Cre mice results in terminal labeling in the CeLC, including in the anterior portion of the CeC where *withdrawal*FOS+ neurons are particularly dense (**Fig. S10b-d**). Our RABV*d*G tracing also revealed monosynaptic inputs to CeLC^PKCδ^ neurons that were primarily located in the elPBN (**Fig. S10e**). To determine whether the MOR+ terminals might synapse on CeLC^PKCδ^ neurons, we coupled RABV*d*G-GFP tracing with *in situ* hybridization to label *Oprm1* mRNA in RABV*d*G-GFP-labeled cells in the PBN (**Fig. S10f-g**). We determined that the majority (42/72 identified cells, roughly 58%) of PBN monosynaptic inputs to CeLC^PKCδ^ neurons express the mu opioid receptor (**Fig. S10g**).

References

1. Sorge RE, Martin LJ, Isbester KA, Sotocinal SG, Rosen S, Tuttle AH, et al. Olfactory exposure to males, including men, causes stress and related analgesia in rodents. Nat Methods. 2014;11.

2. Alipio JB, Brockett AT, Fox ME, Tennyson SS, deBettencourt CA, El-Metwally D, et al. Enduring consequences of perinatal fentanyl exposure in mice. Addiction Biology. 2021;26.

3. Jo AY, Xie Y, Rodriguez A, Sandoval Ortega RA, Creasy KT, Beier KT, et al. VTA μ–opioidergic neurons facilitate low sociability in protracted opioid withdrawal. BioRxiv. 2024. 11 July 2024.

4. Ji G, Neugebauer V. Hemispheric lateralization of pain processing by amygdala neurons. J Neurophysiol. 2009;102.

5. Allen HN, Chaudhry S, Hong VM, Lewter LA, Sinha GP, Carrasquillo Y, et al. A Parabrachial-to-Amygdala Circuit That Determines Hemispheric Lateralization of Somatosensory Processing. Biol Psychiatry. 2023;93.

6. Sadler KE, McQuaid NA, Cox AC, Behun MN, Trouten AM, Kolber BJ. Divergent functions of the left and right central amygdala in visceral nociception. Pain. 2017;158.

7. Friard O, Gamba M. BORIS: a free, versatile open-source event-logging software for video/audio coding and live observations. Methods Ecol Evol. 2016;7.

8. Chaudun F, Python L, Liu Y, Hiver A, Cand J, Kieffer BL, et al. Distinct µ-opioid ensembles trigger positive and negative fentanyl reinforcement. Nature. 2024. 22 May 2024. https://doi.org/10.1038/s41586-024-07440-x.

9. Delfs JM, Zhu Y, Druhan JP, Aston-Jones G. Noradrenaline in the ventral forebrain is critical for opiate withdrawal-induced aversion. Nature. 2000;403:430–434.

10. Corder G, Ahanonu B, Grewe BF, Wang D, Schnitzer MJ, Scherrer G. An amygdalar neural ensemble that encodes the unpleasantness of pain. Science (1979). 2019;363.

11. Chaplan SR, Bach FW, Pogrel JW, Chung JM, Yaksh TL. Quantitative assessment of tactile allodynia in the rat paw. J Neurosci Methods. 1994;53.

12. Clapham DE, Miller C. A thermodynamic framework for understanding temperature sensing by transient receptor potential (TRP) channels. Proc Natl Acad Sci U S A. 2011;108.

13. Godynyuk E, Bluitt MN, Tooley JR, Kravitz A V., Creed MC. An open-source, automated home-cage sipper device for monitoring liquid ingestive behavior in rodents. ENeuro. 2019;6.

14. Kiyokawa Y, Takahashi D, Takeuchi Y, Mori Y. The right central amygdala shows greater activation in response to an auditory conditioned stimulus in male rats. Journal of Veterinary Medical Science. 2016;78.

15. Schindelin J, Arganda-Carreras I, Frise E, Kaynig V, Longair M, Pietzsch T, et al. Fiji: An open-source platform for biological-image analysis. Nat Methods. 2012;9.

16. Carrasquillo Y, Gereau IV RW. Hemispheric lateralization of a molecular signal for pain modulation in the amygdala. Mol Pain. 2008;4.

17. Franklin K, Paxinos G. Paxinos and Franklin’s the Mouse Brain in Stereotaxic Coordinates, Compact. Academic Press, 2019.

18. Stringer C, Wang T, Michaelos M, Pachitariu M. Cellpose: a generalist algorithm for cellular segmentation. Nat Methods. 2021;18.

19. Pachitariu M, Stringer C. Cellpose 2.0: how to train your own model. Nat Methods. 2022;19.

20. Bruno CA, O’Brien C, Bryant S, Mejaes JI, Estrin DJ, Pizzano C, et al. pMAT: An open-source software suite for the analysis of fiber photometry data. Pharmacol Biochem Behav. 2021;201.

21. Chon U, Vanselow DJ, Cheng KC, Kim Y. Enhanced and unified anatomical labeling for a common mouse brain atlas. Nat Commun. 2019;10.

22. Lauridsen K, Ly A, Prévost ED, McNulty C, McGovern DJ, Tay JW, et al. A Semi-Automated Workflow for Brain Slice Histology Alignment, Registration, and Cell Quantification (SHARCQ). ENeuro. 2022;9.

23. Faul F, Erdfelder E, Lang AG, Buchner A. G*Power 3: A flexible statistical power analysis program for the social, behavioral, and biomedical sciences. Behav Res Methods, vol. 39, 2007.

24. Taylor BK, Corder G. Endogenous analgesia, dependence, and latent pain sensitization. Curr Top Behav Neurosci. 2014;20.

25. Lewter LA, Arnold RL, Narosov NB, Dussor G, Kolber BJ. Sex differences in the effects of calcitonin gene-related peptide signaling on migraine-like behavior in animal models: a narrative review. Front Neurol. 2025;16:1603758.

26. Lorsung R, Cramer N, Alipio JB, Ji Y, Han S, Masri R, et al. Sex Differences in Central Amygdala Glutamate Responses to Calcitonin Gene-Related Peptide. J Neurosci. 2025;45.

27. Magee SN, Sereno AC, Herman MA. Sex differences in basal motivated behavior, chronic ethanol drinking, and amygdala activity in female and male mice. Alcohol. 2024;120:85–97.

28. Alvarez-Bagnarol Y, Vendruscolo LF, Marchette RCN, Francis C, Morales M. Neuronal Correlates of Hyperalgesia and Somatic Signs of Heroin Withdrawal in Male and Female Mice. ENeuro. 2022;9.

29. Angst MS, Clark JD. Opioid-induced hyperalgesia: A qualitative systematic review. Acute Pain. 2006;8.

30. Rieb LM, Norman W V., Martin RE, Berkowitz J, Wood E, McNeil R, et al. Withdrawal-associated injury site pain (WISP): A descriptive case series of an opioid cessation phenomenon. Pain. 2016;157.

31. Torres-Rodriguez JM, Wilson TD, Singh S, Torruella-Suárez ML, Chaudhry S, Adke AP, et al. The parabrachial to central amygdala pathway is critical to injury-induced pain sensitization in mice. Neuropsychopharmacology. 2024;49.

32. Jaramillo AA, Brown JA, Winder DG. Danger and distress: Parabrachial-extended amygdala circuits. Neuropharmacology. 2021;198.

33. Li JN, Sheets PL. Spared nerve injury differentially alters parabrachial monosynaptic excitatory inputs to molecularly specific neurons in distinct subregions of the central amygdala. Pain. 2020;161.

34. Liu S, Ye M, Pao GM, Song SM, Jhang J, Jiang H, et al. Divergent brainstem opioidergic pathways that coordinate breathing with pain and emotions. Neuron. 2022;110.
